# Fetal hemoglobin induction in azacytidine responders enlightens methylation patterns related to blast clearance in higher-risk MDS and CMML

**DOI:** 10.1186/s13148-024-01687-x

**Published:** 2024-06-15

**Authors:** Theodora Chatzilygeroudi, Vasiliki Chondrou, Ruben Boers, Stavroula Siamoglou, Katerina Athanasopoulou, Evgenia Verigou, Joost Gribnau, Spyridon Alexis, Vassiliki Labropoulou, Alexandra Kourakli, George P. Patrinos, Argyro Sgourou, Argiris Symeonidis

**Affiliations:** 1 School of Health Sciences, Faculty of Medicine, Hematology Division, University of Patras, Patras, Greece; 2 Division of Hematological Malignancies, Department of Oncology, Sidney Kimmel Comprehensive Cancer Center, Johns Hopkins University, Baltimore, MD USA; 3 Biology Laboratory, School of Science and Technology, Hellenic Open University, Patras, Greece; 4 Department of Developmental Biology, Faculty of Medicine and Health Sciences, Erasmus University Medical Center, Rotterdam, The Netherlands; 5 Laboratory of Pharmacogenomics and Individualized Therapy, Department of Pharmacy, School of Health Sciences, University of Patras, University Campus, Rio, Patras, Greece; 6 Department of Genetics and Genomics, College of Medicine and Health Sciences, United Arab Emirates University, Al Ain, Abu Dhabi, UAE; 7 Zayed Center for Health Sciences, United Arab Emirates University, Al Ain, Abu Dhabi, UAE

**Keywords:** Myelodysplastic syndromes, Prognosis, Fetal hemoglobin, ZBTB7A, Methylation patterns

## Abstract

**Background:**

As new treatment options for patients with higher-risk myelodysplastic syndromes are emerging, identification of prognostic markers for hypomethylating agent (HMA) treatment and understanding mechanisms of their delayed and short-term responses are essential. Early fetal hemoglobin (HbF) induction has been suggested as a prognostic indicator for decitabine-treated patients. Although epigenetic mechanisms are assumed, responding patients’ epigenomes have not been thoroughly examined. We aimed to clarify HbF kinetics and prognostic value for azacytidine treated patients, as well as the epigenetic landscape that might influence HbF re-expression and its clinical relevance.

**Results:**

Serial HbF measurements by high-performance liquid chromatography (*n* = 20) showed induction of HbF only among responders (*p* = 0.030). Moreover, HbF increase immediately after the first azacytidine cycle demonstrated prognostic value for progression-free survival (PFS) (*p* = 0.032, HR = 0.19, CI 0.24–1.63). Changes in methylation patterns were revealed with methylated DNA genome-wide sequencing analysis (*n* = 7) for *FOG-1, RCOR-1*, *ZBTB7A* and genes of the NuRD-complex components. Targeted pyrosequencing methodology (*n* = 28) revealed a strong inverse correlation between the degree of γ-globin gene (*HBG2)* promoter methylation and baseline HbF levels (*p* = 0.003, *r*_s_ =  − 0.663). A potential epigenetic mechanism of HbF re-expression in azacytidine responders was enlightened by targeted methylation analysis, through hypomethylation of site -53 of *HBG2* promoter (*p* = 0.039, *r*_s_ =  − 0.504), which corresponds to MBD2-NuRD binding site, and to hypermethylation of the CpG326 island of *ZBTB7A* (*p* = 0.05, *r*_s_ = 0.482), a known HbF repressor. These changes were associated to blast cell clearance (*p*_HBG2_ = 0.011, *r*_s_ = 0.480/*p*_ZBTB7A_ = 0.026, *r*_s_ = 0.427) and showed prognostic value for PFS (*p*_ZBTB7A_ = 0.037, HR = 1.14, CI 0.34–3.8).

**Conclusions:**

Early HbF induction is featured as an accessible prognostic indicator for HMA treatment and the proposed potential epigenetic mechanism of HbF re-expression in azacytidine responders includes hypomethylation of the γ-globin gene promoter region and hypermethylation of the CpG326 island of *ZBTB7A.* The association of these methylation patterns with blast clearance and their prognostic value for PFS paves the way to discuss in-depth azacytidine epigenetic mechanism of action.

**Graphical abstract:**

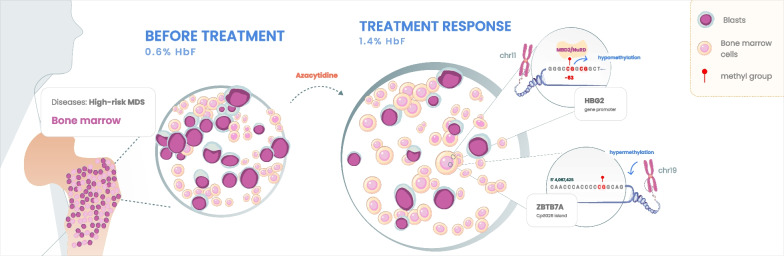

**Supplementary Information:**

The online version contains supplementary material available at 10.1186/s13148-024-01687-x.

## Background

Hypomethylating agents (HMAs), azacytidine (AZA) and decitabine (DAC), remain the mainstay of treatment for higher-risk myelodysplastic syndromes (HR-MDS) and for a subset of secondary acute myeloid leukemia (AML) patients in the last two decades [1, 2]. The same treatment option is administered to transplant-ineligible patients with unfavorable chronic myelomonocytic leukemia (CMML), inducing similar response rates [3, 4]. A substantial response is only achieved by 30–50% of treated patients [5, 6] after a median of 4–5 months post-treatment initiation [7]. However, many aspects of the induced hypomethylating effect, as well as additional potential activity on the cellular genetic and epigenetic landscape, remain unclear [8]. The relatively short-term duration of responses (18–20 months for patients with MDS [9]) and the inevitable development of resistance to HMAs have widely been reported. Thus, the identification of prognostic factors [10–13] and a deeper understanding of the underlying mechanisms, directing the development of response or resistance to HMAs, are of substantial clinical importance.

Recent studies in HR-MDS or AML patients have indicated that epigenetically modulated γ-globin gene expression, resulting in increase of fetal hemoglobin (HbF) production, could represent a potential dynamic biomarker for the outcome, following DAC treatment [14]. Indeed, the early induction of HbF, estimated by high-performance liquid chromatography (HPLC), an accessible technique widely used, has been shown to have prognostic value for DAC-treated MDS or AML patients [14]. Moreover, the prognostic significance of pretreatment HbF levels for the overall survival of patients receiving AZA [15] or DAC [16] has also been demonstrated, possibly reflecting altered DNA methylation across γ-globin gene promoter and higher de novo sensitivity to HMAs [14]. A similar hypothesis was assumed for pretreatment methylation levels of long interspersed element *(LINE)-1* [17]. *LINE-1* is member of the retroelement family of LINEs that constitute 10% of CpG sites of our genome [18], and thus represents an established marker for global DNA methylation [19]. Recently, the importance of HbF for MDS prognosis was also expanded to lower-risk MDS [20], further underscoring its clinical significance.

AZA is known to induce γ-globin gene expression and consequently, to increase HbF levels from its use to sickle cell anemia patients [21]. A recent study demonstrated an increase of HbF-containing red blood cells in patients receiving AZA, indicating HbF levels as a potential biomarker of treatment efficacy [22]. Even though an epigenetic mechanism of HbF induction is hypothesized, methylation status of the γ-globin gene promoter and responding patients’ epigenomes have not been examined. Understanding the underlying mechanism(s), beneath HbF expression in responding patients could enlighten potential, as yet unknown pathways of HMA action and highlight an accessible biomarker for HMA treatment response, with high clinical relevance.

The multifactorial epigenetic mechanism of HbF silencing (Additional file 1) includes several key players, the main of which are B-cell leukemia/lymphoma 11A (BCL11A) and leukemia/lymphoma-related factor (LRF), encoded by the *ZBTB7A* gene [23]. Both of these central HbF regulators are also implicated in oncogenesis [24–26]; however, LRF is epigenetically modulated and regulates γ-globin chain expression independently of BCL11A, via the nucleosome remodeling and deacetylase (NuRD)-associated pathway [27]. NuRD is one of the main chromatin remodeling complexes, constituted of many subunits [23–28] (Additional file 1), and plays a pivotal role in many cancer types [29]. Moreover, friend of GATA-1 (FOG-1) gene, established to be required for the “GATA switch” in terminal erythroblast maturation [30], cooperates with GATA-1 to induce γ-globin chain gene silencing [23]. Repressor element-1 silencing factor corepressor-1 (CoREST) encoded by the *RCOR-1* gene is part of the complex that associates lysine-specific demethylase 1 (LSD1) with BCL11A, to mediate its strong silencing activity [23] (Additional file 1). Recently*, RCOR-1* gene expression was shown to be essential for AML cell survival in different cell lines [31]. This dual role of HbF modifying factors, implicated both, in normal hematopoiesis and cancer biology, renders the unraveling of HbF re-expression mechanism in HR-MDS patients treated with HMA an appealing puzzle.

In this prospective cohort study, we investigated HbF kinetics and the prognostic significance of its early increase in AZA-treated HR-MDS patients. Additionally, we aimed to clarify the underlying epigenetic landscape that contributes to increased HbF expression, such as alterations in γ-globin gene promoter methylation status, by using methylated DNA genome-wide sequencing analysis (MeD‑seq) combined with targeted pyrosequencing methodology. Finally, to determine the clinical relevance of our results, we performed analysis of the outcomes, according to AZA treatment response, overall (OS) and progression-free survival (PFS).

## Methods

### Patients, sample collection and response evaluation

Thirty-three patients with HR-MDS, CMML or chemotherapy-naive secondary AML with ≤ 25% bone marrow blasts, received AZA by standard scheduling (75mg/m^2^ × 7 days, 28-day cycle) at the University Hospital of Patras. All consecutively treated patients between December 2020 and August 2023 were included (no selection criteria were applied). Marrow complete response (mCR) was defined according to the IWG 2006 criteria, as myeloblasts ≤ 5% and decreased ≥ 50% over pretreatment, at first evaluation of response (5–7 cycles of AZA) [32]. Patients were assessed for hematological improvement of one or more lineages (HI), according to recent revised criteria for clinical trials [33]. Since in our specific cohort all patients exhibiting HI were mCR responders, we also conducted a separate analysis for hematological responders (HI-R = mCR + HI), to feature changes seen only in patients with hematological response to AZA. Non-responders (NR) were defined as patients not achieving mCR at first evaluation of response. Hematological-non-responders (HNR) were defined as patients without HI, even when they achieved mCR. PFS and OS were estimated since treatment initiation. Disease progression was defined as > 25% relative increase in blasts in peripheral blood or bone marrow, compared to baseline.

Bone marrow aspirate samples were collected before treatment initiation and at the evaluation of response (after 5–7 cycles). Whole genomic DNA was extracted using phenol: chloroform: isoamyl acid in 25:24:1 ratio (Sigma-Aldrich Pty Ltd, An affiliate of Merck KGaA, Darmstadt, Germany). One patient was evaluated after the 3rd AZA cycle, due to suspected disease progression.

The study was approved by the University General Hospital of Patras Ethics Committee (approval number 33807/24.12.2020). All patients provided their written informed consent according to the Declaration of Helsinki, being informed about both, clinical and translational investigations.

### Hemoglobin quantification by HPLC

Peripheral blood HbF levels were measured by HPLC at the Laboratory of Hematology Division of the University Hospital of Patras, using the Variant II hemoglobin testing system, at baseline, and following 1, 2, 4 and 6 cycles of treatment with AZA (HbF_0_, HbF_1_, HbF_2_, HbF_4_ and HbF_6_, respectively).

### MeD-seq and processing FastQ files

MeD-seq analyses were carried out as previously described [34, 35] for a seven patients’ cohort of our AZA-treated patients. This separate cohort consisted of four mCR responders and three NR patients. The DNA methylation-dependent enzyme LpnPI (New England Biolabs) was used to digest a total of 14 samples, resulting in fragments of around 31–32 bp, that contained methylated regions. The fragments were processed by ThruPlex DNA–seq 96D kit (Rubicon Genomics Ann Arbor) to generate an Illumina NGS library for each sample. Subsequently, the products were purified on Pippin HT system with DNA gel cassettes for 100–250bp (Sage Science) and single-ended sequencing was carried out on Illumina HiSeq2500 systems with 50 bp read length (Illumina). Bcl2FastQ conversion software was used to demultiplex dual indexed samples. FASTQ files were processed as previously described [34] to output only methylated DNA fragments, using specified scripts in Python to trim Illumina adapters and filter data based on LpnPI restriction site. After mapping, accepted reads to human genome 38 (hg38) using Bowtie2, LpnPI site scores were used, to generate read count scores for transcription start sites (1 kb prior and 1 kb post TSS), as well as CpG islands and gene bodies (1 kb post TSS until TES). ENSEMBL (www.ensembl.org) was used to download specifics of genes and CpG islands. The gene annotation version for hg38 used was “hg38.GRCh38.79.” The final dataset of significant differentially methylated regions (DMRs) was obtained, using the Chi-square test on read counts of samples pre- and post-treatment (*p* < 0.05) and the Benjamini–Hochberg procedure to overcome the multiple testing problem (FDR and q values < 0.05). From this final dataset of DMRs, we selected changes located within genetic loci, encoding for molecules implicated in γ-globin gene expression [23, 36], such as *BCL11A, ZBTB7A, GATA-1, FOG-1*, etc. (Additional file 2, Table S1). Genes which demonstrated altered methylation patterns in more than one patient were included in our analysis.

### Targeted methylation analysis

**Fig. 1:**
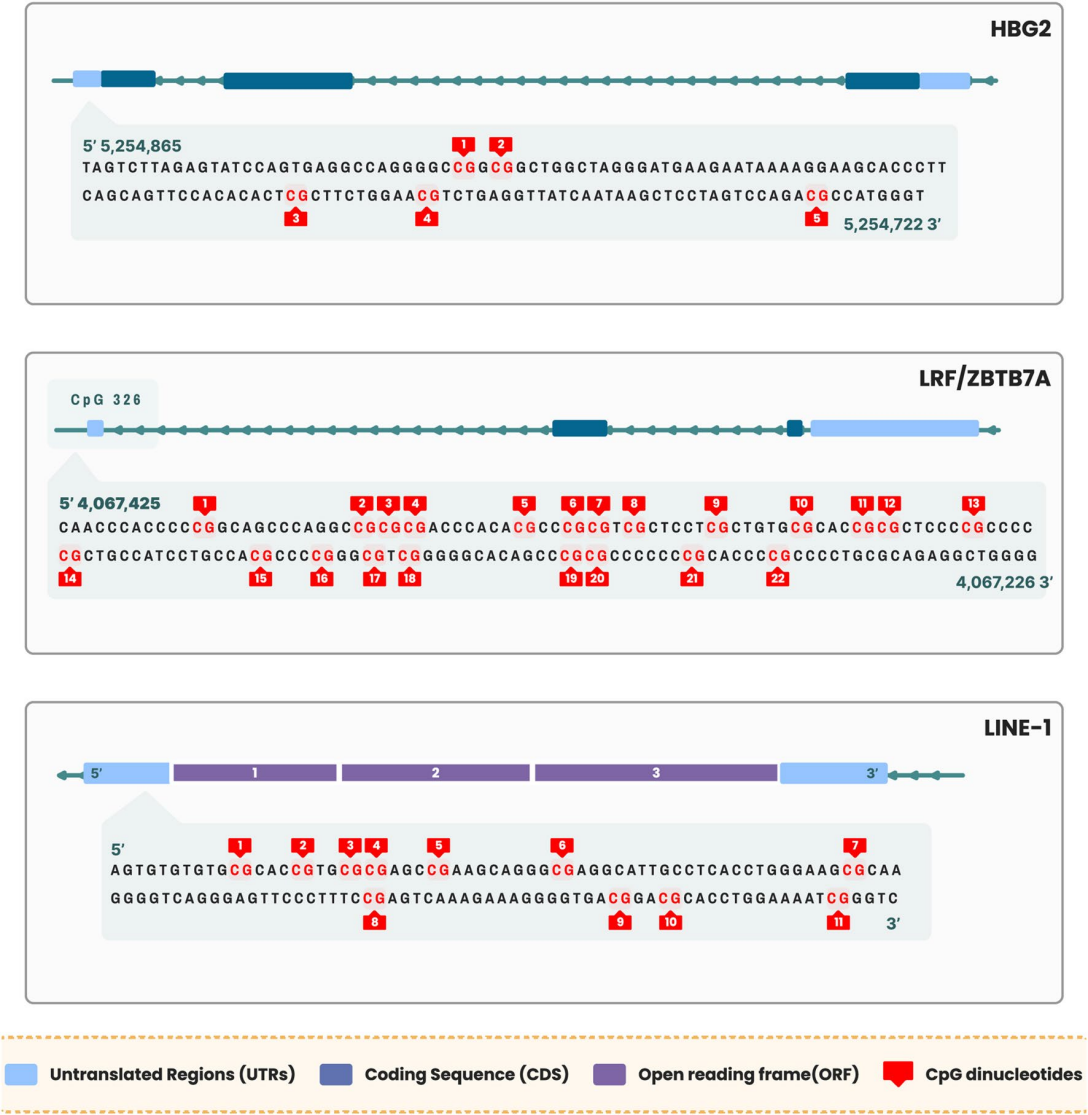
Schematic representation of HBG2 and ZBTB7A/LRF gene loci and the LINE-1 region. The CG dinucleotides analyzed by pyrosequencing are presented. HBG2: hemoglobin subunit gamma 2, ZBTB7A: zinc finger and BTB domain containing 7A, LRF: leukemia/lymphoma-related factor, LINE-1: long interspersed element-1

In twenty-eight patients’ DNA samples, pyrosequencing CpG assay methodology was used to identify methylation levels of the γ-globin gene (*HBG2*) promoter and the CpG326 island of *ZBTB7A*/LRF gene (Fig. 1). We also analyzed methylation levels of *LINE-1* (Fig. 1), known to be a marker of global methylation in cancer [19]. Methylation levels were measured in bone marrow whole genomic DNA samples before treatment initiation and at the first evaluation of response (5–7 cycles of AZA).

Genomic DNA extraction was followed by bisulfite conversion of unmethylated cytosines, existing as CG dinucleotides, to uraciles with EpiTech® Bisulfite Kit (QIAGEN GmbH, Hilden Germany). Regions of interest were amplified with PyroMark® PCR Kit (QIAGEN GmbH, Hilden, Germany) from the bisulfite converted DNA product. Pyrosequencing reactions were performed with the PyroMark Q24 MDx technology (QIAGEN GmbH, Hilden, Germany). Sequencing and PCR primers were designed with the PyroMark Assay Design Software, version 2.0 (QIAGEN GmbH, Hilden, Germany) and are shown in Additional file 2 (Table S2). Quality controls were conducted in every run set for bisulfite conversion efficiency and the methylation status of all sequences was confirmed in duplicate analysis, confirming reproducibility and validation of the method.

### Statistical analysis

Aspiring to pursue 80% power and 95% confidence level, and estimating from previous MDS studies [14] that differences pre- and post-ΗΜΑ (paired samples) would be 0.8 (%) at 2–4 cycles of HMA, and that standard deviation would be around 0.8, we resulted in a sample size of 10 patients. To assess different groups of patients, we aimed to study 20 patients.

As a first approach to explore HbF induction significance over time, in the whole 20-patient cohort and within different subpopulations, nonparametric Friedman one-way repeated measure analysis was used. As a further post hoc analysis, paired samples Wilcoxon signed-rank test was applied, to identify significant changes between the different time points (at baseline and after 1, 2, 4 and 6 cycles of AZA treatment). Distributions of HbF values between responders, non-responders or other subgrouping were compared using Student’s t test or Mann–Whitney U test, according to normality of the data. The mCR and HI-R responders were compared to the rest of the patients (NR and HNR, respectively). Fisher’s exact test was employed to investigate differences between various groups, concerning categorical variables (i.e., classification based on a designated threshold). One patient was excluded from our analysis as an outlier, due to very high HbF level before treatment (HbF 11.4%), indicating another potentially underlying pathophysiology.

Targeted methylation data from the 28-patient cohort were analyzed as per each GG site and mean methylation. Paired samples t test or Wilcoxon test was used to identify significant changes post-treatment, according to normality of the data. Correlation analysis with Spearman correlation coefficient (*r*_s_) was conducted to investigate the association between HbF levels or their increase with target genes methylation levels/changes and other variables (e.g., IPSS-R).

The Kaplan–Meier method was used to estimate distributions of overall and progression-free survival. Log-rank test was used to assess the prognostic significance and a Cox model to estimate the hazard ratio (HR) and its 95% confidence interval (95% CI).

The normality of the data was evaluated using the Kolmogorov–Smirnov test. The level of significance was set at *α* = 0.05. Statistical analyses were performed by using IBM SPSS 28 Statistical Package.

## Results

### Significant HbF induction was documented in azacytidine responders

HbF levels pre- and post-1, -2, -4 and -6 cycles of AZA treatment were assessed for 20 patients with HR-MDS or CMML. Baseline disease characteristics and demographic features of our complete cohort are demonstrated in Table 1, and no differences between responders and non-responders were observed. After eliminating one patient with very high HbF levels before treatment, this 19-patient cohort included 11 mCR (7 of them HI-R) and 8 NR patients.

**Table 1:** Patients' characteristics

Patient group	All patients *n* = 33	mCR *n* = 17	NR *n* = 16
Age (years, median/range)	71 (58–88)	71 (58–88)	70 (60–87)
Sex			
Male	30	16	14
Female	3	1	2
Diagnosis			
MDS-MLD	4	3	1
MDS-EB I	13	7	6
MDS-EB II	9	4	5
CMML	3	1	2
MDS-EB II/AML	4	2	2
Bone marrow blasts (%, median/range)	9.5 (1–25)	9 (1–25)	10 (1–22.5)
Hb (g/dl, median/range)	7.83 (6.2–11.8)	7.8 (6.2–11.8)	7.82 (7.1–10.4)
WBC (× 10^9^, median/range)	3.21 (0.76–32.8)	3.16 (0.76–24.1)	3.26 (1.19–32.8)
ANC (× 10^9^, median/range)	1.12 (0.07–26.3)	0.96 (0.07–13.8)	1.28 (0.51–26.3)
PLT (× 10^9^, median/range)	71.8 (13–357)	65 (13–284)	105 (15.8–357)
LDH (U/dl)	192 (119–823)	196 (119–415)	189 (143–823)
CRP	0.62 (0.13–15.86)	0.53 (0.13–8.48)	0.7 (0.18–15.86)
β2-microglobulin	3.4 (1.2–7.9)	3.4 (2.2–7.8)	3.5 (1.2–7.9)
IPSS-R	5.5 (2.0–9.0)	5.5 (2.5–7.0)	5.5 (2.0–9.0)

mCR: marrow responders, NR: non-responders, MDS: myelodysplastic syndrome, AML: acute myeloid leukemia, MDS-MLD: MDS with multilineage dysplasia, MDS-EBI: MDS with excess blasts I (5–9% blasts), MDS-EBII: MDS with excess blasts II (10–19% blasts), MDS-EBII/AML: MDS-EBII suspicious for AML progression, Hb: hemoglobin, WBC: white blood cells, ANC: absolute neutrophil count, PLT: platelets, LDH: lactate dehydrogenase, CRP: c-reactive protein, IPSS-R: Revised International Prognostic Scoring System

**Fig. 2:**
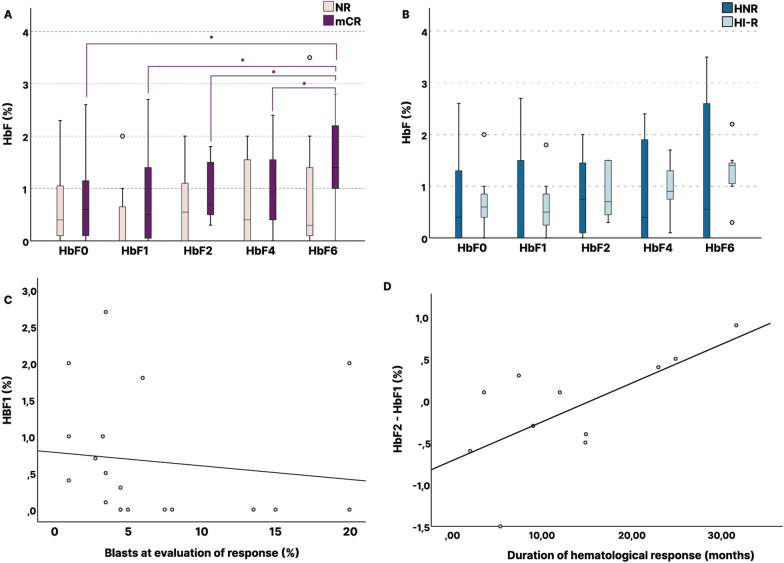
Early fetal hemoglobin increase is related to blast clearance and prognostic of progression-free survival. **A** Fetal hemoglobin (HbF) kinetics in marrow responders (mCR) (*n* = 11), and non-responding (NR) patients (*n* = 8). *p* < 0.05(*). **B** HbF kinetics in hematological responders (HI-R = mCR + HI) (*n* = 7) and non-responders (NHR) patients. **C** HbF after 1 cycle of azacytidine (HbF1) is related to blast clearance (blasts = 0.78–0.02*HbF1). **D** Increase of HbF levels between the 1st and 2nd treatment cycle is strongly associated with longer duration of hematological response in HI-R patients (*p* = 0.026, *r*_s_ = 0.665) [(HbF2-HbF1) =  − 0.72 + 0.05*(duration of HI)]

Analyzing HbF kinetics during AZA treatment, among recruited patients, we observed an increasing trend to median values of 0.7%, 0.8% and 1.1% after 2, 4 and 6 cycles of treatment, respectively, compared to median pretreatment HbF levels of 0.6% (*p* = 0.101) (Additional file 3, Figure S1). When separate analysis was performed, non-responders (NR) displayed a decreasing trend of HbF levels during treatment (*p* = 0.380), especially in HbF1 (*p* = 0.114), although not statistically significant. On the contrary, mCR responders, showing a baseline median HbF of 0.6%, gradually increased it to 0.7%, 0.9% and 1.4% after 2, 4 and 6 cycles of treatment (*p* = 0.030). Post hoc analysis revealed a significant increase in HbF at the evaluation of response (following 6 cycles of AZA treatment) in mCR responders (*p* = 0.019) (Fig. 2A). The most prominent HbF induction in this patient subpopulation was observed between the 4th and 6th cycle of treatment (*p* = 0.047) (Fig. 2A). Conducting the analysis according to HI-R classification, the same trend toward HbF induction was observed, although it did not reach statistical significance (*p* = 0.065) (Fig. 2B).

Exploring the relationship between baseline characteristics [baseline cytopenias and IPSS-R, IPSS-Molecular (M) scoring] and HbF induction, no specific correlation was noted (*p* > 0.1).

### Early HbF increase is associated with blast cell clearance and has a predictive value for PFS

Investigating the link between HbF induction and response to AZA, we observed a tendency indicating an association between HbF levels ≥ 1% at the initial evaluation of AZA response (HbF6) and the attainment of mCR (*p* = 0.058). The threshold of 1% has also been set as a predictive marker of response in previous studies [14]. Moreover, as expected from HbF kinetics in responders, higher HbF values following the first cycle of AZA treatment were associated with significantly lower bone marrow blast cell percentage at first response evaluation (*p* = 0.030, *r*_s_ = -0.498, Fig. 2C). In addition, the increase of HbF levels between the 1st and 2nd treatment cycle was strongly associated with longer duration of hematological response in HI-R patients (*p* = 0.026, *r*_s_ = 0.665) (Fig. 2D).

**Fig. 3:**
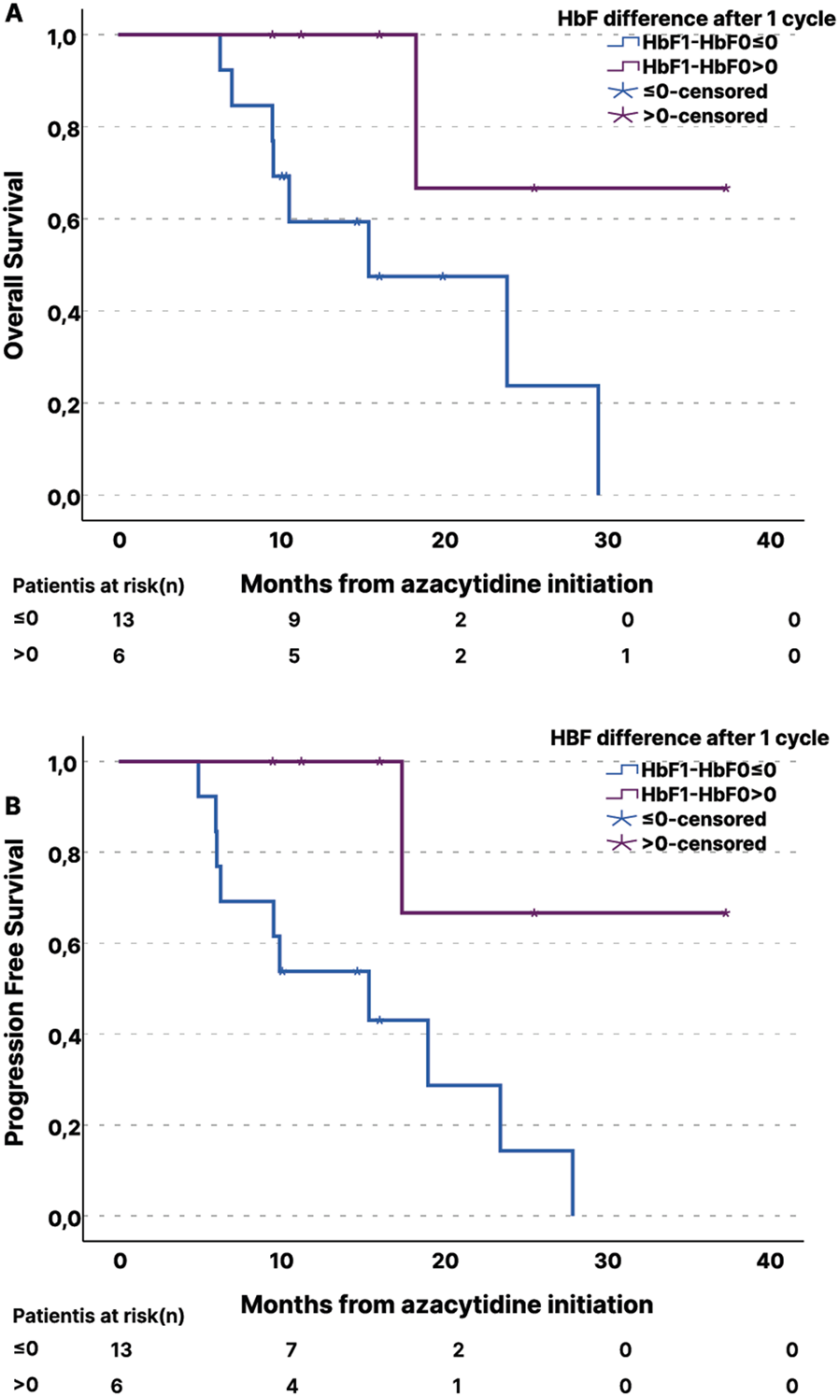
Kaplan–Meier analyses of overall survival (**A**) and progression-free survival (**B**) according to whether HbF increased or not after one cycle of azacytidine. Patients in whom HbF was increased after the first cycle of AZA, OS and PFS, were longer, compared to those of patients, who exhibited decreased or stable HbF: median OS 19.7 vs 14.1 months and median PFS 19.5 vs 13 months (*p*_OS_ = 0.065, HR = 0.17, CI 0.22–1.4, *p*_PFS_ = 0.032, HR = 0.19, 95% CI 0.24–1.63)

Assessing the potential predictive value of early HbF induction, among six patients, in whom HbF was early increased, after the first cycle of AZA, we found that both, OS and PFS, were longer, compared to those of patients, who exhibited decreased or stable HbF: median OS 19.7 vs 14.1 months (*p* = 0.065, HR = 0.17, CI 0.22–1.4) and median PFS 19.5 vs 13 months (*p* = 0.032, HR = 0.19, 95% CI 0.24–1.63), respectively (Fig. 3A, B).

Even though larger cohorts have demonstrated that baseline HbF has prognostic value for the outcome of HMA treatment [15, 16], in our HR-MDS patient cohort, baseline HbF levels (HbF_0_ ≥ 1%) did not correlate with the achievement of mCR (*p* = 1.00) and a predictive value for OS or PFS was not demonstrated (*p*_OS_ = 0.354, *p*_PFS_ = 0.937). This result is limited by the fact that only six of our patients had HbF_0_ ≥ 1%.

### Hypomethylation of the γ-globin gene promoter post-AZA treatment in responders is related to HbF expression and blast cell clearance

The full 28-patient cohort of HR-MDS and CMML patients, consisting of 15 mCR responders (10 of them HI-R) and 13 NR patients, displayed median methylation levels of the γ-globin gene promoter sequence of 78.9% and 78.2% pre- and post-AZA treatment (*p* = 0.139), respectively. Baseline levels (pre-AZA) were similar between mCR and NR (*p* = 0.206) patients, with a baseline mean methylation level of 78.2% and 80%, respectively (Additional file 3, Figures S2-S3).

**Fig. 4:**
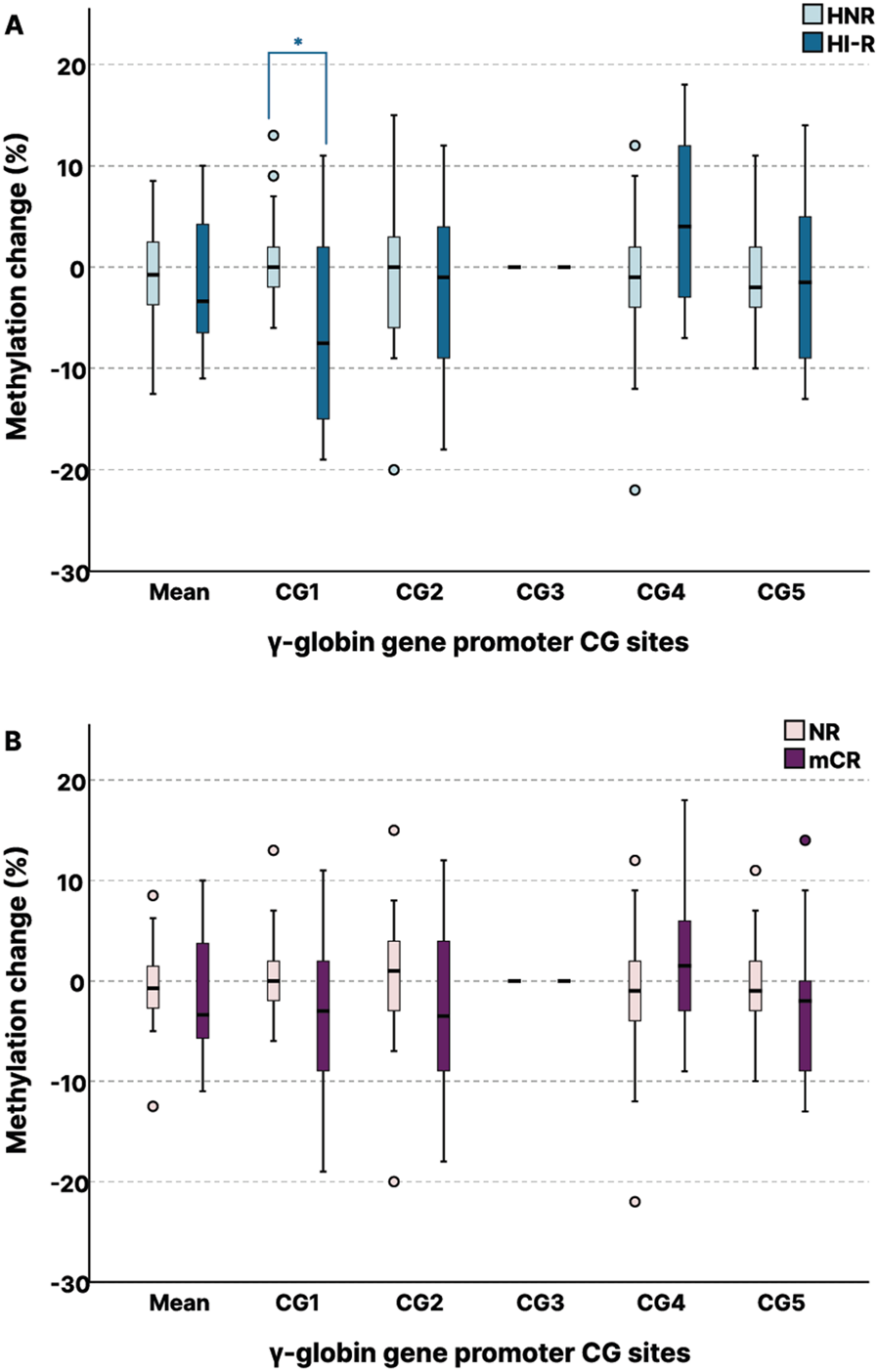
Gamma-globin promoter methylation status alteration in azacytidine responders. **A** Reduction in methylation of CG1 of the γ-globin gene (*HBG2*) promoter is greater in HI-R responders (*n* = 10) than NHR patients (*p* = 0.03). **B** The same trend was observed for mCR responders (*n* = 15) compared to non-responders (NR) (*p* = 0.08). mCR = marrow response (blasts ≤ 5% and decreased ≥ 50% over pretreatment and HI-R = marrow response and hematological improvement of at least one lineage according to revised IWG criteria 2018. p* < 0.05

**Fig. 5:**
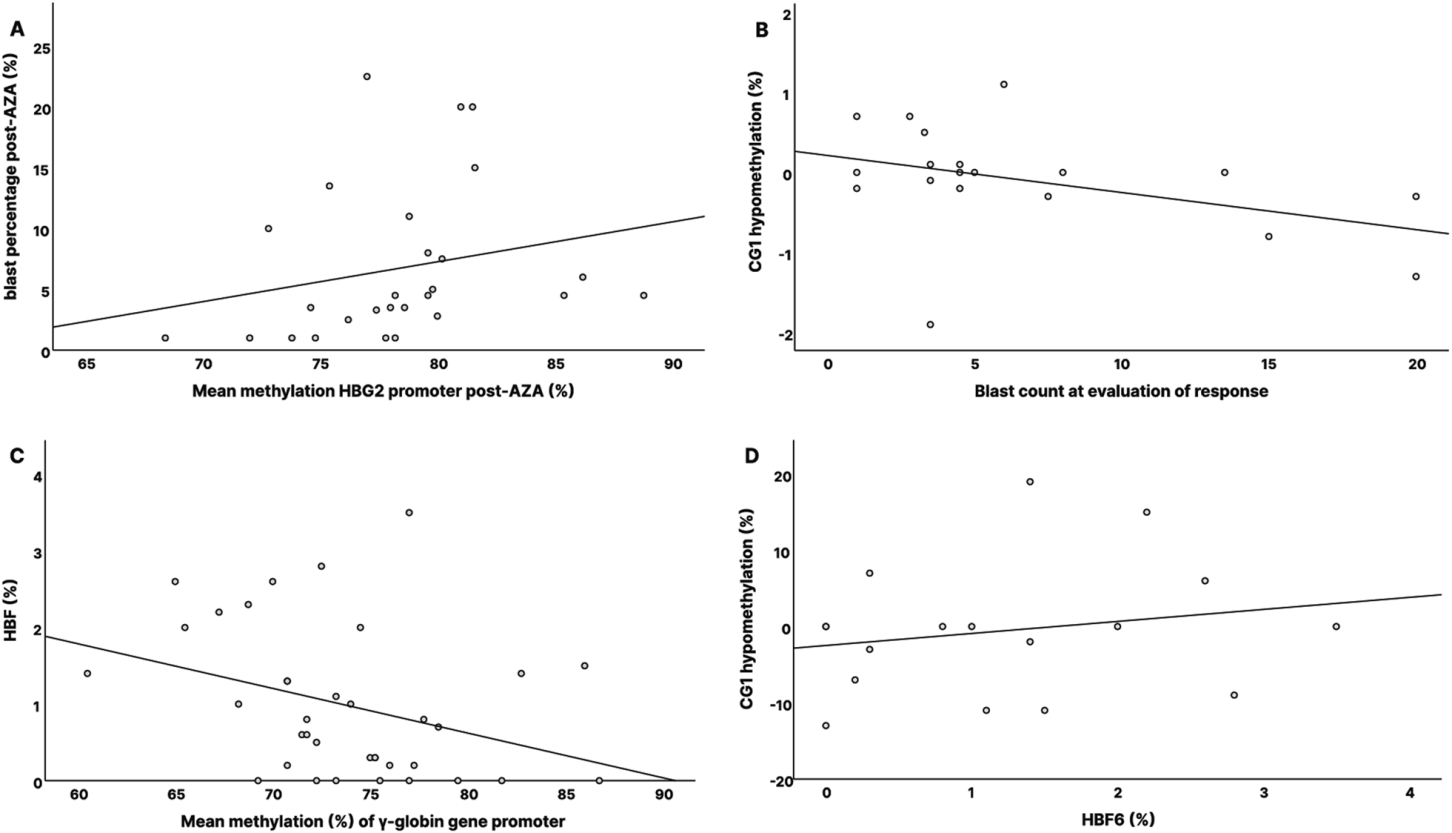
Gamma-globin promoter methylation status alteration correlates to fetal hemoglobin expression and blast clearance. **A** Mean methylation levels of *HBG2* promoter post-AZA correlates to blast count (*p* = 0.011, *r*_s_ = 0.482). **B** Hypomethylation at CG1 relates to less blast count at evaluation of response (*p*_1_ = 0.005, *r*_s1_ = 0.527). **C** Mean methylation of the *HBG2* promoter is reversely related to HbF levels [HbF = 5.29–0.06*(HG2 promoter methylation)] (*p* = 0.016, *r*_s_ =  − 0.398). **D** Hypomethylation at CG1 relates to higher HbF levels at evaluation of response (HbF6) (*p* = 0.039, *r*_s_ =  − 0.504)

Figure 1 illustrates *HBG2* gene with the upstream promoter sequence and highlights CG sites (CG1-CG5) studied. Among both, responders and NRs, CG3 remained stably 100% methylated pre- and post-AZA treatment. Responders (mCR) showed a trend of hypomethylation in CG1, CG2 and CG5 sites of the γ-globin gene promoter (Fig. 1), with mean methylation level reduction to 77% post-AZA treatment (*p*_mCR_ = 0.159) (Additional file 3, Figure S2). Moreover, methylation of CG1 was significantly more reduced in HI-Rs (*p*_1HI-R_ = 0.03) compared to HNRs, and the same trend was observed for mCRs (*p*_1mCR_ = 0.08) compared to NRs (Fig. 4A, B). Lower overall- and CG5 mean methylation levels (Additional file 3, Figure S7), as well as hypomethylation at CG1 of the γ-globin gene promoter region post-AZA treatment, were correlated with lower bone marrow blast cell percentage (*p* = 0.011, *r*_s_ = 0.482/*p*_5_ = 0.005, *r*_s5_ = 0.528/*p*_1_ = 0.005, *r*_s1_ = 0.527, respectively) (Fig. 5A, B). Accordingly, patients with stable or reduced methylation levels at CG1 post-AZA treatment exhibited median OS of 23.9 months and PFS of 23.5 months, compared to 10.5 and 9.9 months, respectively, of patients with increase in CG1 methylation post-AZA. Nevertheless, prognostic significance was not reached (*p*_OS_ = 0.759, HR = 1.22, 95% CI 0.34–4.3/*p*_PFS_ = 0.393, HR = 0.63, 95% CI 0.22–1.83).

Examining all simultaneous methylation and HbF level measurements pre- and post-AZA (HbF0 and HbF6), we found that methylation status of the γ-globin gene promoter demonstrated an inverse association to HbF expression (*p* = 0.016, *r*_s_ = -0.398, Fig. 5C). A significant negative correlation with HbF expression was additionally demonstrated, separately for CG1 and CG5 methylation levels (*p*_1_ = 0.042, *r*_s_ = -0.341/ and *p*_5_ = 0.005, *r*_s_ = -0.461, respectively) (Additional file 3, Figure S4, S5). When analysis was restricted to baseline (pre-AZA treatment) values, the correlation was stronger (*p* = 0.003, *r*_s_ = -0.663), whereas the association was lost when analyzing only the post-treatment values (*p* = 0.467, *r*_s_ = -0.189) (Additional file 3, Figure S6). At disease evaluation post-AZA treatment, HbF expression was associated with hypomethylation of the CG1 site of the promoter (*p* = 0.039, *r*_s_ = -0.504), which corresponds to site -53 of the *HBG2* promoter (Fig. 5D).

### MeD-seq analysis reveals altered methylation patterns of HbF modifying genes post-AZA treatment

Changes in methylation of HbF modifying genes were found in five out of seven AZA-treated patients (Additional file 4, Table S3). Overall, and as expected, most sites were hypomethylated post-AZA treatment, with significant fold change (most percentage values < 1, Table 2). Gene specifics of human genome 38 and CpG island sites were retrieved from UCSC (University of California Santa Cruz) genome browser (https://genome.ucsc.edu/) and alterations in methylation are schematically shown in Additional file 5.

**Table 2:** Significant differentially methylated regions (DMRs) of fetal hemoglobin regulating genes after treated with azacytidine

Patient	A		B		C		E		F	
HMA	Azacytidine		Azacytidine		Azacytidine		Azacytidine		Azacytidine	
Response	CR		mCR + HI		mCR		NR		NR	
Gene	DMRs: genomic region / percentage of methylation change after AZA									
*ZFPM-1 (FOG-1)*	chr16:88,460,664–88,462,117	2.4	chr16:88,493,412–88,493,681	0.75	chr16:88,506,877–88,507,081	0.32	chr16:88,488,889–88,488,947	0.11	chr16:88,488,348–88,489,194	0.43
	chr16:88,465,688–88,465,867	3.9			chr16:88,533,202–88,535,122	0.24	chr16:88,492,229–88,494,836	0.51	chr16:88,491,479–88,491,617	1.43
	chr16:88,468,258–88,468,492	0.97			chr16:88,502,152–88,502,376	0.22	chr16:88,533,052–88,533,207	0.63	chr16:88,492,229–88,494,515	0.4
	chr16:88,479,813–88,480,155	3.6							chr16:88,500,830–88,501,498	0.6
	chr16:88,492,583–88,494,346	0.6							chr16:88,501,941–88,502,056	0.51
	chr16:88,494,422–88,494,451	0.4							chr16:88,502,199–88,502,754	0.51
	chr16:88,499,186–88,499,386	1.18							chr16:88,531,325–88,533,288	0.4
	chr16:88,523,032–88,523,155	0.75								
	chr16:88,523,855–88,524,702	2.23								
	chr16:88,526,936–88,527,054	0.9								
	chr16:88,527,976–88,528,122	0.86								
*RCOR-1*	chr14:102,676,134–102,676,253	1.11	chr14:102,591,589–102,593,707	0.4	No changes		chr14:102,676,123–102,678,174	0.49	chr14:102,676,123–102,678,028	0.46
	chr14:102,676,410–102,676,546	0.76								
	chr14:102,676,700–102,677,531	0.63								
	chr14:102,677,565–102,677,611	0.8								
	chr14:102,677,684–102,677,826	0.72								
	chr14:102,683,924–102,684,520	2.3								
*ZBTB7A*	chr19:4,059,818–4,059,925	0.93	No changes		chr19:4,044,919–4,045,156	0.37	chr19:4,053,881–4,055,321	0.53	chr19:4,043,303–4,043,304	0.57
	chr19:4,060,102–4,060,965	1.75								
	chr19:4,062,674–4,065,060	4.47*								
	chr19:4,065,187–4,065,271	0.93								
*MTA2*	chr11:62,601,631–62,603,368	2.6	No changes		chr11:62,600,986–62,603,019	0.16	No changes		No changes	
	chr11:62,601,461–62,601,591	0.93								
*MTA1*	chr14:105,421,147–105,423,091	2.47	No changes		chr14:105,441,559–105,441,781	2.61	chr14:105,464,044–105,465,176	0.46	chr14:105,450,201–105,450,507	0.37
									chr14:105,465,846–105,466,723	0.36
									chr14:105,466,815–105,467,406	0.58
									chr14:105,470,070–105,470,729	0.36
*MBD3*	No changes		No changes		chr19:1,580,400–1,580,631	0.06	chr19:1,584,231–1,585,730	0.55	chr19:1,585,160–1,585,343	0.43
									chr19:1,584,231–1,585,094	0.45
									chr19:1,585,547–1,585,615	0.79
*BCL11A*	No changes		chr2:60,552,567–60,554,567	0.12	chr2:60,494,686–60,496,686	0.13	No changes		No changes	
			chr2:60,549,461–60,555,644	0.07						

DMRs: Differentially methylated regions, AZA: azacytidine, ZFPM-1: zinc finger protein multitype 1, FOG-1: friend of GATA-1 (FOG-1), RCOR-1: REST corepressor-1, ZBTB7A: zinc finger and BTB domain containing 7A, MTA1/2: metastasis-associated proteins 1 or 2, MBD3: methyl-CpG binding domains 3, BCL11A: B-cell lymphoma/leukemia 11A, mCR: marrow response (blasts ≤ 5% and decreased ≥ 50% over pretreatment), HI-R: marrow response and hematological improvement of at least one lineage according to revised IWG criteria 2018, NR: no response. Genomic region chr19:4,062,674–4,065,060 including part of the CpG326, 5’ upstream of ZBTB7A is annotated (*). Values > 1 indicate hypermethylation and values < 1 hypomethylation

Concerning NuRD-complex components, CpG16 and CpG85 islands of the methyl-CpG binding domain paralog MBD3 were found with hypomethylated regions in non-responding patients (Table 2). Another component of the NuRD complex, *metastasis-associated protein (MTA)2*, was found to have developed methylation changes in two responding patients. Patient A exhibited hypermethylated regions mainly within the CpG207 and TSS region, whereas patient C demonstrated hypomethylation in a broader length, including CpG207 along with sequences in the *MTA2* coding regions. Two responding patients exhibited hypermethylated intron regions of *MTA1*, whereas hypomethylation of CpG89, CpG38 and CpG28 islands, located across exons/introns of *MTA1*, was observed in non-responders (Additional file 5).

*FOG-1 or ZFPM-1 (zinc finger protein multitype 1)* is an 83,752 bp gene in length, which contains several CpG islands. Our best responding patient (A), who achieved a complete marrow response and normalized all hematopoietic cell lineages (CR), exhibited hypermethylation patterns mainly in gene coding areas of *FOG-1*, whereas hypomethylated DMRs were predominantly found in regions containing CpG islands. All hypermethylated DMRs included non-coding gene regions. Two responding patients, but not achieving CR, and all the non-responding patients had mainly hypomethylated DMRs that included mainly CpG islands. CpG20 island of *FOG-1* was hypomethylated in four out of five patients (Additional file 5).

Regarding *RCOR-1* gene, although changes were found among both, responders and non-responders, most of them pertained to non-CpG intron regions, and only one responding patient (patient B) demonstrated hypomethylation of the CpG184 island, located upstream of *RCOR-1* exon1. *BCL11A* demonstrated methylation alterations in two patients, either in CpG island regions or not (Additional file 5).

Genomic region chr19:4,062,674–4,065,060 including part of the CpG326, 5’ upstream of *ZBTB7A*, known for its regulatory effect on LRF and subsequent HbF expression in thalassemic patients [37], showed the greater hypermethylation effect in the best responding patient A (annotated with “*” in Table 2). Although hypermethylated regions of *ZBTB7A* were only encountered in this responder, non-responded patients exhibited several hypomethylated regions including CpG17, CpG154 and exon3 (Additional file 5). These results led us to examine with pyrosequencing the methylation profile of the CpG326 region, previously studied and known to influence LRF expression and HbF regulation [37].

### Hypermethylation of CpG326 island upstream of *ZBTB7A* gene is associated with HbF expression, blast clearance and PFS in ΑΖΑ-responders

**Fig. 6:**
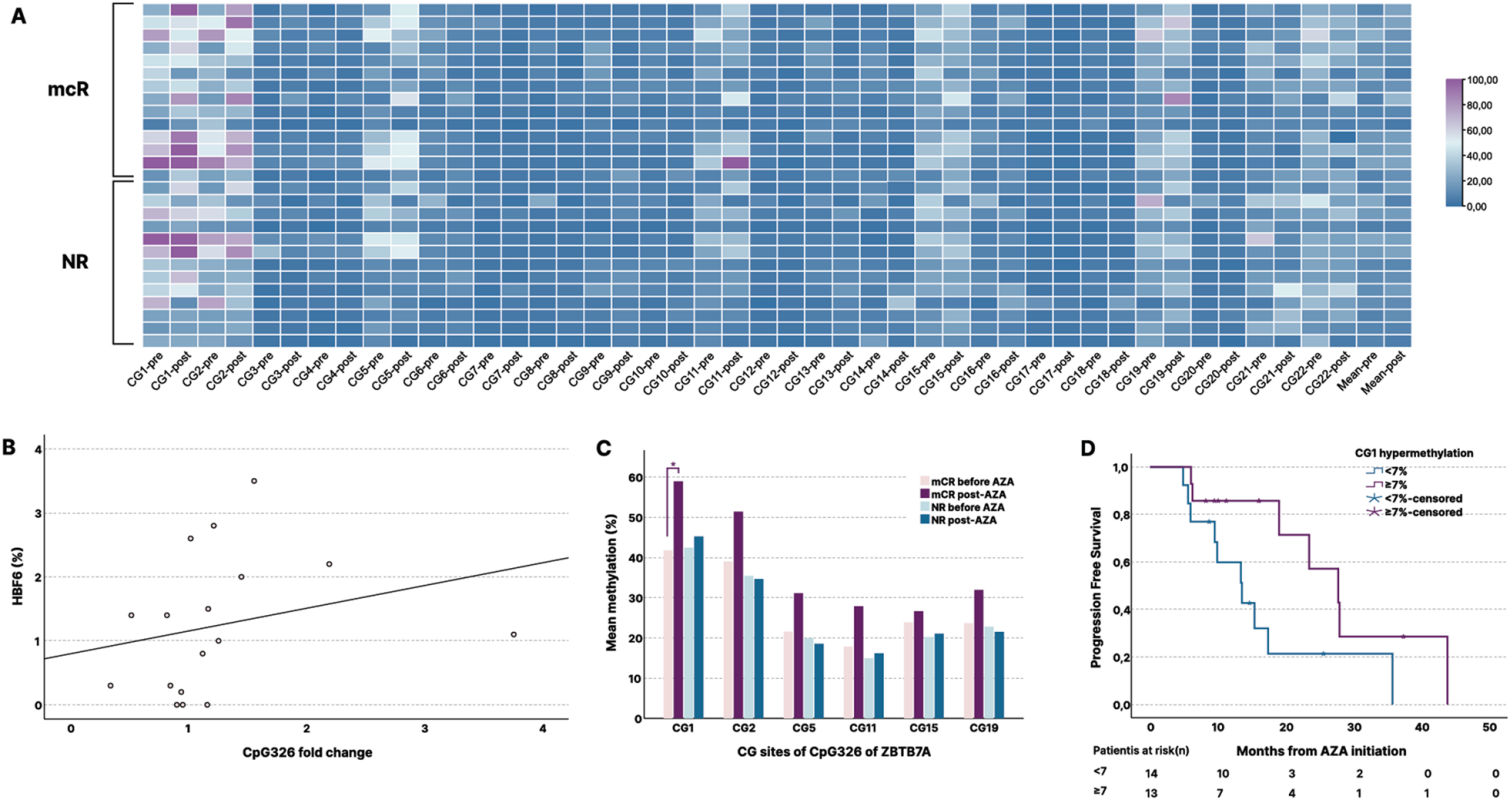
Changes in methylation patterns of the CpG326 of the *ZBTB7A* after azacytidine treatment. **A** Heat map of methylation patterns of CG sites of the CpG326 of *ZBTB7A* pre- and post-azacytidine treatment in marrow responders (mCR) (*n* = 15) and non-responders (NR) (*n* = 13). The heat map was created with TBtools. **B** Increase in methylation of CpG326 is related with HbF at evaluation of response to azacytidine (HbF6). **C** CG sites of CpG326 with hypermethylation in marrow responders. Significant increase in methylation of CG1.*p* < 0.05(*). **D** Kaplan–Meier analysis for progression-free survival according to having above or equal to median hypermethylation (≥ 7%) of CpG326 (*p* = 0.037 HR = 1.14, 95% CI 0.34–3.8)

Methylation analysis of the CpG326 island of *ZBTB7A* showed significant increase in methylation of CG1 from 42.1% to 52.3% (*p* = 0.041) and a trend of hypermethylation at CG2, CG5, CG11 and CG19 (Fig. 6A). In contrast to what we observed before AZA treatment initiation, when no association between CpG326 methylation status and HbF values was found, there was a significant correlation between HbF values and the increase of CpG326 methylation after 6 cycles of AZA treatment (*p* = 0.05, *r*_s_ = 0.482) (Fig. 6B). Moreover, HbF values ≥ 1% were correlated with the mean degree of hypermethylation of the CpG326 island (*p* = 0.047, *r*_s_ = -0.481). In separate analysis, a similar trend was also observed for CG1, CG3, CG5, CG11 and CG22 (*p*_1_ = 0.077, *p*_3_ = 0.052, *p*_5_ = 0.087, *p*_11_ = 0.061 and *p*_22_ = 0.060, respectively).

When analysis was restricted to mCR responders, there was a significant increase of methylation at CG1 (*p*_1_ = 0.020) and a trend of hypermethylation at CG5 and CG11 (*p*_5_ = 0.069, and *p*_11_ = 0.079, respectively), whereas changes were not significant for NR patients (Fig. 6C). Interrogating relation of the methylation status of CpG326 island post-AZA treatment to blast cell clearance, a significant correlation was noted (*p* = 0.026, *r*_s_ = 0.427) (Additional file 4, Figure S8). Accordingly, patients displaying hypermethylation of CG1 of median degree or above (≥ 7%) were grouped together. Their mean PFS was estimated at 18.8 months, compared to 13.9 months of patients exhibiting < 7% hypermethylation or hypomethylation and the log rank test indicated a prognostic value (*p* = 0.037, HR = 1.14, 95% CI 0.34–3.8) (Fig. 6D).

### *LINE-1* methylation levels assessment reveals global hypomethylation effects in AZA responders

Investigation of methylation changes of the *LINE-1* region (Fig. 1) revealed hypomethylation only among responders (*p*_mCR_ = 0.012), and specifically at CG1 and CG3 sites (*p*_1_ = 0.001, *p*_3_ = 0.028, respectively). On the contrary, NR patients demonstrated hypermethylation of CG6 (*p* = 0.044) (Additional file 4, Figure S9). This internal validation evaluated the global hypomethylating effect of AZA between the different response groups of the present study. Although methylation reduction was greater in mCR responders (*p* = 0.047), pretreatment methylation levels of *LINE-1* were at equal levels between different response groups (*p* = 0.348) (Additional file 4, Figure S10).

Looking closely at our patients’ cytogenetic and molecular next-generation sequencing data, we identified two mCR patients with karyotypic abnormalities and one NR with *TET2* mutation at diagnosis, who had repeated their molecular/karyotypic testing at response evaluation. At this time point, which corresponds also to the time of methylation pyrosequencing analysis post-AZA treatment, these molecular/karyotypic abnormalities persisted, implying the persistence of the clonal cell population. Moreover, the 2 mCR patients exhibited hypermethylated CpG326 with hypomethylated *LINE-1* (CG1), whereas the NR patient exhibited hypomethylated CpG326, along with hypermethylated γ-globin promoter and *LINE-1* (CG1) (Additional file 4, Figure S11). This further supports the hypothesis that the observed hypo- and hypermethylation patterns are epigenetic changes on the clonal hematopoietic cells.

## Discussion

The continuous spectrum between myelodysplasia and acute leukemia is a unique field of cancer biology where gradually evolving oncogenesis (leukemogenesis) meets hematopoiesis [38]. The association between HbF induction and hypermethylation of the CpG326 island of *ZBTB7A* gene, coding for one of the main HbF repressors, that was observed in AZA responders, is not merely a proposed mechanism for γ-globin gene re-expression in MDS patients, treated with HMA. This observation also opens the way to an in-depth discussion regarding HMA mechanism of action. Although the name and inhibitory activity on DNMTs implies a global hypomethylating effect, previous studies have shown hypermethylated regions post-HMA treatment [39]. Previously, a stable clonal architecture of MDS and CMML patients responding to AZA treatment has been shown, indicating that the cytological response appears not to be associated with a reduction of the mutated clone, but rather to a change in its epigenetic profile [40]. This was indeed, verified in selected patients of our cohort with available cytogenetic/molecular data pre- and post-AZA treatment. Thus, our results indicate that clonal bone marrow progenitors of MDS patients responding to AZA treatment exhibit this hypermethylation pattern of CpG326 of *ZBTB7A*, that is correlated with increased HbF levels.

Investigated in many cancer types, LRF (ZBTB7A) has either oncogenic or tumor suppressor functions, highlighting its ability to act differently, depending on the existing epigenetic landscape [24]. Among the other ZBTB factors with a clear role in hematopoiesis [41], LRF regulates lineage fate by blocking monocytic differentiation and promoting granulopoiesis [42]. LRF has been found to positively affect transcription of NF-κB [24], which is a key player in MDS progression toward secondary AML [43], and its silencing is known to promote apoptosis in cancer cells [24]. Conversely, LRF has recently been shown to prevent RUNX1-RUNX1T1-mediated clonal expansion of human CD34 + cells, thus suggesting a protective effect against developing AML with (8;21) translocation [42]. Evidently from the above analysis, LRF appears as an appealing target for investigation in MDS, especially in the context of hypomethylating agent treatment.

Furthermore, as a “friend of GATA,” *FOG-1* is known to promote mostly megakaryocytic and erythroid commitment, while it concomitantly suppresses myeloid progenitor specification via NuRD recruitment [44, 45]. In our results, although hypomethylated regions were typically identified in CpG islands, hypermethylated regions were mostly found in *FOG-1* coding gene body areas. In previous reports, rebound gene body methylation, following HMA treatment, resulted in increased gene expression, a phenomenon dependent of the DNMT3B activity [46], a methyltransferase not inhibited by HMAs. Given its role in hematopoiesis, questions arise from *FOG-1* possible overexpression, in response to AZA. For instance, one might wonder for its potential relation to resistant neutropenia in patients exhibiting hematological improvement or in non-responding patients. Further research is clearly needed to decode and clarify the methylation/expression changes and the role of *FOG-1* on AZA responders.

In our study, we demonstrated an inverse association of the γ-globin gene promoter methylation status with HbF baseline expression in HR-MDS patients. However, the hypothesis that methylation status of the γ-globin gene locus could represent a marker for de novo sensitivity to HMAs [14, 16] was not proven, since baseline methylation levels did not differ between the differently responded groups. Moreover, as data from larger MDS/AML cohorts indicated [14, 16], pretreatment HbF levels were slightly lower in the NR group of our HR-MDS cohort (Fig. 2A). However, neither did it differ significantly, compared to responders nor did it exhibit prognostic value for PFS. Similarly, pretreatment *LINE-1* methylation status did not correlate to AZA response. On the contrary, higher HbF levels following AZA treatment, were related to hypomethylation of -53 site of the γ-globin gene promoter and were associated with a favorable response.

Notably, *MTA1* and *MTA2*, as well as *MBD3*, were found to be hypomethylated in NR patients and their possible overexpression was in accordance with the decrease in HbF observed in this group of patients. The role of these genes in cancer biology [47] raises questions about their significance in HMA resistance. Even though the association of the NuRD-complex components’ genes methylation changes with HbF levels was not clarified by our study, NuRD-associated silencing could be potentially linked to our results. In previous research, MBD2 knock-down has been shown to exhibit more prominent effects on HbF expression than MBD3 in human adult erythroid cells [48]. Moreover, MBD2 binds with high affinity to site -53/-50 of *HBG2* promoter to direct γ-globin silencing in concert with BCL11A [49]. Its highly preferential binding to methylated CpG-rich DNA [49, 50] renders the hypomethylation of site -53 in responders as a potential mechanism of reversing γ-globin gene silencing. However, further mechanistic studies are warranted to prove this conclusion.

The perspective of using HbF measured by HPLC as a prognostic marker for response, following HMA treatment was proposed after studying HbF kinetics in DAC-treated MDS and AML patients [14]. However, determining HbF kinetics and its prognostic importance for AZA-treated patients was a gap that required to be filled. Our cohort data demonstrated a gradual increase of HbF levels, from a baseline value of 0.6% to 1.4%, after 6 cycles of AZA treatment, exclusively among responders. We observed that following the first month/cycle, patients who are about to become unresponsive to treatment drop their HbF levels, whereas patients, who are going to respond, preserve or increase them. Although limited by the rather small sample size, our study showed that the increase in HbF levels immediately after the first cycle of AZA treatment demonstrated prognostic value for patient’s PFS. Given that in the MeD-seq results hypomethylation of some HbF repressor genes was observed, this initial drop in HbF levels among non-responding patients could be explained.

In the study of Stomper et al. [14], HbF levels were measured every 6 weeks, during DAC treatment, due to differences in treatment scheduling. In MDS patients, median levels of 1.9% were achieved in DAC responders after 12 weeks of treatment, whereas in our AZA-treated cohort only 0.9% of HbF was reached after 4 cycles (~ 16 weeks). Moreover, elevated HbF levels after 2 cycles (12 weeks) of treatment with DAC in MDS/AML patients were also related to blast clearance at 6 months. HbF levels as early as 4 weeks were not measured in the study of Stomper et al. [14], so the early decrease of HbF in NR patients cannot be compared for the two drugs. Overall, DAC appears to induce higher levels of HbF during the first months of treatment [14]. Apart from differences in the two drugs specific mechanism of action, these differences could be attributed to inhomogeneity of the patient cohorts, or to different time points of peripheral blood samples collection from the time of actual HMA administration (i.e., not at the end of each cycle), as well as to the use of different laboratory instruments for HPLC estimation.

In our study, we aimed to justify kinetics and prognostic value of HbF in a very specific group of patients (HR-MDS and CMML), but the rarity of such samples did not permit us to test a larger cohort. Moreover, although we are grateful to our patients for permitting us to collect bone marrow samples at frequent time intervals, we did not manage to study mRNA expression of genes with variable methylation patterns, due to restricted cell content of the bone marrow aspirates. Finally, HbF estimation by HPLC could have been biased by the parallel administration of red blood cell transfusions in some of our patients [51], and thus, HbF quantification would be more accurate if measured as far from transfusion as possible, a parameter that was not taken into consideration in our study.

In practical terms, to effectively use early HbF induction as a prognostic marker for response to HMA treatment, and to appropriately select patients for clinical trials, including HMA-based regimens combined with novel and emerging treatment options [52], HbF measuring techniques should be standardized. Additionally, with the introduction of venetoclax-HMA combination for MDS patients [53], there may be a need to reassess the significance of HbF determination. Therefore, evaluation in bigger patient cohorts, consisting exclusively of MDS or AML patients, is essential to validate the relevance of findings from this study in real-world scenarios.

## Conclusions

In conclusion, in this study we showed that HbF is only induced in AZA responders and that early HbF increase could be a potential prognostic marker for PFS in HR-MDS. Moreover, we propose an epigenetic mechanism of HbF re-expression in AZA responding HR-MDS patients, through the hypomethylation of site -53 of the γ-globin gene promoter, which is a binding site of MBD2-NuRD, and the simultaneous hypermethylation of the CpG326 island of *ZBTB7A* gene (a known HbF suppressor); a mechanism that needs further clarification. Lastly, we demonstrated altered gene methylation patterns of components of the HbF silencing mechanism, following AZA treatment and their role in response to AZA or AML progression remain open questions.

### Supplementary Information

The multifactorial epigenetic mechanism of HbF silencing.

Supplementary methodology tables.

Supplementary figures_1.

Supplementary tables and figures_2.

Graphical presentation of significant differentially methylated regions (DMRs) of HbF regulating genes in high-risk MDS patients after treated with azacytidine (UCSC genome browser was used).

## Data Availability

FastQ raw files of the methylated DNA genome-wide sequencing analysis are available under BioProject accession number PRJNA1075483 (https://www.ncbi.nlm.nih.gov/bioproject/1075483). Further information about the specified scripts in Python used for MeD-seq analysis is available upon request (r.g.boers@erasmusmc.nl). Additional data available upon reasonable request (argiris.symeonidis@yahoo.gr).

## Abbreviations

AML: Acute myeloid leukemia
AZA: Azacytidine
BCL11A: B-cell leukemia/lymphoma 11A
CI: Confidence interval
CMML: Chronic myelomonocytic leukemia
CoREST: Repressor element-1 silencing factor corepressor-1
CR: Complete remission
DAC: Decitabine
DMR: Differentially methylated region
FOG-1: Friend of GATA-1
HBG2: Hemoglobin subunit gamma 2
HbF: Fetal hemoglobin
HI: Hematological improvement
HI-R: Hematological response
HMA: Hypomethylating agent
HPLC: High-performance liquid chromatography
HR: Hazard ratio
HR-MDS: Higher-risk myelodysplastic syndromes
LINE-1: Long interspersed element-1
LRF: Leukemia/lymphoma-related factor
LSD1: Lysine-specific demethylase 1
MBD: Methyl-CpG binding domain
mCR: Marrow complete response
MeD-seq: Methylated DNA genome-wide sequencing analysis
MTA: Metastasis-associated protein
NR: No response/non-responder
NuRD: Nucleosome remodeling and deacetylase
OS: Overall survival
PFS: Progression-free survival
*r*_s_: Spearman correlation coefficient
UCSC: University of California Santa Cruz
ZBTB7A: Zinc finger and BTB domain containing 7A
ZFPM-1: Zinc finger protein multitype 1
