# Supplementary material for: Fetal hemoglobin induction in azacytidine responders enlightens methylation patterns related to blast clearance in higher-risk MDS and CMML
Source: Clin Epigenetics. 2024 Jun 15;16:79. doi: 10.1186/s13148-024-01687-x (PMC11180405; doi:10.1186/s13148-024-01687-x)
Supplement: Supplementary file 1 — The multifactorial epigenetic mechanism of HbF silencing. [file 13148_2024_1687_MOESM1_ESM.pdf]

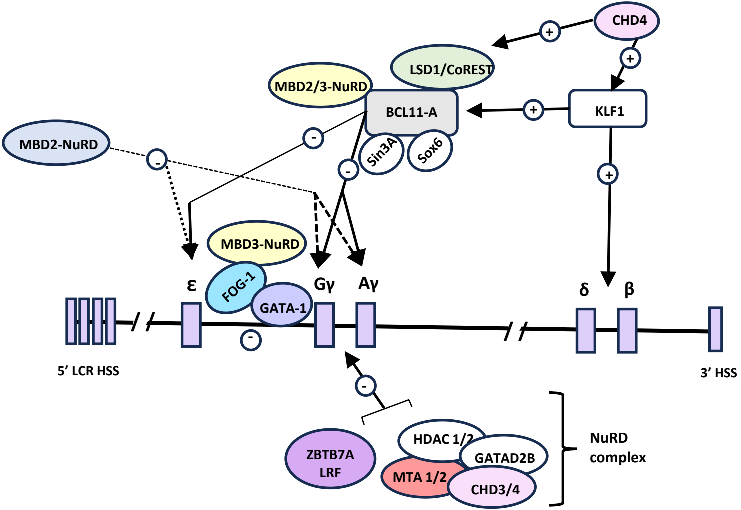

BCL11A: B-cell lymphoma/leukemia 11A (BCL11A), LRF/ZBTB7A: Leukemia/lymphoma-related factor, NuRD: Nucleosome Remodeling and Deacetylase complex, MBD2/3: Mehtyl CpG Binding Domains 2 or 3, CHD3/4: Chromodomain Helicase DNA Binding Proteins 3 or 4, MTA1/2: Metastasis-Associated Proteins 1 or 2, HDAC1/2: Histone Deacetylases 1 or 2, GATA-1: GATA Binding Protein 1, FOG-1: Friend of GATA-1 (FOG-1), CoREST: Repressor element-1 silencing factor corepressor-1, LSD1: Lysine-specific demethylase 1, Sin3A: Paired amphipathic helix protein Sin3a, Sox6: SRY-Box Transcription Factor 6, KLF1: Erythroid Krüppel-Like Factor.
