# Supplementary material for: Fetal hemoglobin induction in azacytidine responders enlightens methylation patterns related to blast clearance in higher-risk MDS and CMML
Source: Clin Epigenetics. 2024 Jun 15;16:79. doi: 10.1186/s13148-024-01687-x (PMC11180405; doi:10.1186/s13148-024-01687-x)
Supplement: Supplementary file 2 — Supplementary methodology tables. [file 13148_2024_1687_MOESM2_ESM.pdf]

Fetal hemoglobin induction in azacytidine responders enlightens methylation patterns related to blast clearance in higher-risk MDS and CMML.

Theodora Chatzilygeroudi, Vasiliki Chondrou, Ruben Boers, Stavroula Siamoglou, Katerina Athanasopoulou, Evgenia Verigou, Joost Gribnau, Spyridon Alexis, Vassiliki Labropoulou, Alexandra Kourakli, George P. Patrinos, Argyro Sgourou, Argiris Symeonidis

Additional file 2: Supplementary methodology tables

Table S1. Genes of main fetal hemoglobin expression regulators.

Gene	Genetic Region
ZBTB7A	chr19: 4,043,303 - 4,066,899
BCL11A	chr2: 60,457,679 - 60,553,658
KLF1 enhancer region	chr19: 12,884,422 - 12,887,201
NF-E2	chr12: 54,292,111 - 54,301,015
GATA-1 (NF-E1, Ery-1 and GF-1)	chrX: 48,786,590 - 48,794,311
GATA-2	chr3: 128,479,427-128,493,201
ZFPM1 (FOG-1)	chr16: 88,453,280 - 88,537,031
SP1	chr12: 53,380,176 - 53,416,446
SOX6	chr11: 15,966,449 - 16,476,388
c-kit ligand (KL)	chr13: 33,016,423 - 33,066,143
Tal-1	chr1: 47,216,291 - 47,232,225
NF-E4 (N46W)	chr7: 102,973,492 - 102,988,847
MBD-2	chr18: 54,151,606 - 54,224,669
MBD-3	chr19: 1,573,596 - 1,592,865
CDH3	Chr16: 68,645,310 - 68,700,292
CHD4 (Mi2β)	chr12: 6,570,082 - 6,607,379
PRMT1	chr19: 49,677,152 - 49,688,447
CHTOP (friend of PRMT1)	chr1: 153,634,066 - 153,646,306
RCOR1	chr14: 102,592,649 - 102,730,561
PRMT5	chr14: 22,920,529 - 22,929,376
MTA1	chr14: 105,419,827 - 105,470,729
MTA2	chr11: 62,593,214 - 62,601,865
Sin3A	chr15: 75,370,933 - 75,455,783
HBG1	chr11: 5,248,269 - 5,249,857
HBG2	chr11: 5,253,188 - 5,254,781
HDAC1	chr1: 32,292,083 - 32,333,626
HDAC2	chr6: 113,933,028 - 113,971,148
KDM1A (LSD1)	chr1: 23,019,468 - 23,083,689

LRF/ZBTB7A: Leukemia/lymphoma-related factor, BCL11A: B-cell lymphoma/leukemia 11A, KLF1: Erythroid Krüppel-Like Factor, NF-E2: Nuclear Factor, Erythroid 2, GATA-1: GATA Binding Protein 1, GATA-2: GATA Binding Protein 2, ZFPM-1: Zinc finger protein multitype 1, FOG-1: Friend of GATA-1 (FOG-1), SP1: Specificity protein 1, SOX6: SRY-box transcription factor

6, KL: c-kit ligand, TAL-1: T-cell acute leukemia 1, NF-E4: Nuclear Factor, Erythroid 4, MBD2/3: Mehtyl CpG Binding Domains 2 or 3, CHD3/4: Chromodomain Helicase DNA Binding Proteins 3 or 4, PRMT1: Protein Arginine Methyltransferase 1, CHTOP: chromatin target of PRMT1, RCOR-1: REST corepressor 1, PRMT5: Protein Arginine Methyltransferase 5, MTA1/2: Metastasis-Associated Proteins 1 or 2, SIN3A: SIN3 Transcription Regulator Family Member A, HBG1: Hemoglobin Subunit Gamma 1, HBG2: Hemoglobin Subunit Gamma 2, HDAC1/2: Histone Deacetylases 1 or 2, KDM1A: (K)-specific demethylase 1A, LSD1: Lysine-specific demethylase 1.

Table S2. PCR primers used for targeted pyrosequencing methodology.

Assay	Gene	Sequence	Primer
CpG assay	*ZBTB7A/LRF*	5' GTAGATTTTTTTGTGTTAAGGA 3'	Forward
		5' AACAAACCCCCAACCTCTAC 3'	5'biotinylated reverse
		5' GGGATTTTTATAGTTTTATTTTTAA 3'	Forward/Pyrosequencing
	HBG2	5' ATGGTGGGAGAAGAAAATTAGTT 3'	Forward
		5' TCTCCTCCAACATCTTCCACATTCA 3'	5'biotinylated reverse
		5' ATAGTTTTAGAGTATTTAGTGA 3'	Forward/Pyrosequencing
	LINE1	5' AAGTAAGTTTGGGTAATGG 3'	Forward
		5' AACAACTCCCATCTACAACTCCC 3'	5'biotinylated reverse
		5' GGGTGGGAGTGAT 3'	Forward/Pyrosequencing
