# Supplementary material for: Fetal hemoglobin induction in azacytidine responders enlightens methylation patterns related to blast clearance in higher-risk MDS and CMML
Source: Clin Epigenetics. 2024 Jun 15;16:79. doi: 10.1186/s13148-024-01687-x (PMC11180405; doi:10.1186/s13148-024-01687-x)
Supplement: Supplementary file 3 — Supplementary figures_1. [file 13148_2024_1687_MOESM3_ESM.pdf]

Fetal hemoglobin induction in azacytidine responders enlightens methylation patterns related to blast clearance in higher-risk MDS and CMML.

Theodora Chatzilygeroudi, Vasiliki Chondrou, Ruben Boers, Stavroula Siamoglou, Katerina Athanasopoulou, Evgenia Verigou, Joost Gribnau, Spyridon Alexis, Vassiliki Labropoulou, Alexandra Kourakli, George P. Patrinos, Argyro Sgourou, Argiris Symeonidis

Additional file 3: Supplementary figures_1

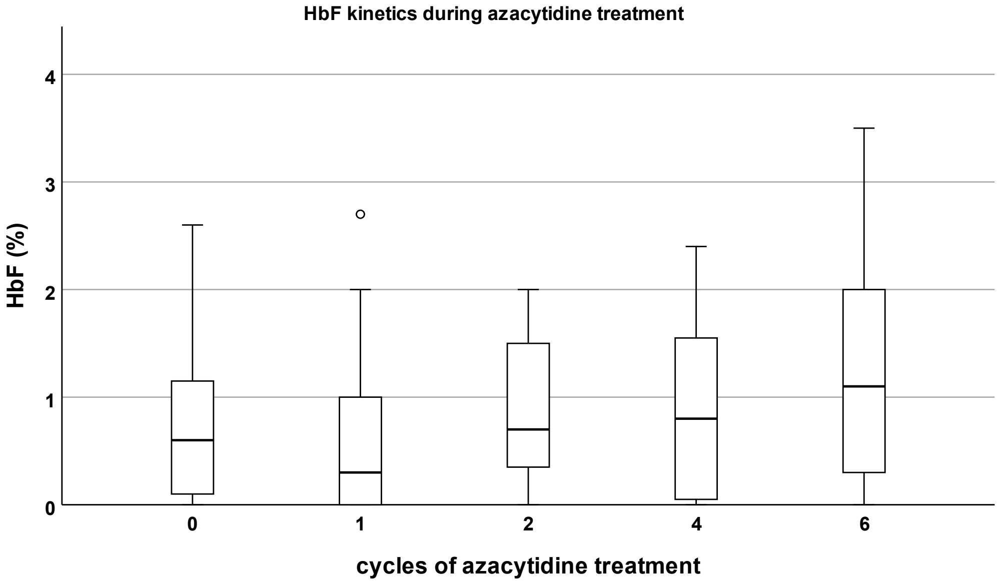

Figure S1. HbF kinetics in the whole 19 patients' cohort over time (after 1, 2,4 and 6 cycles of azacytidine treatment).

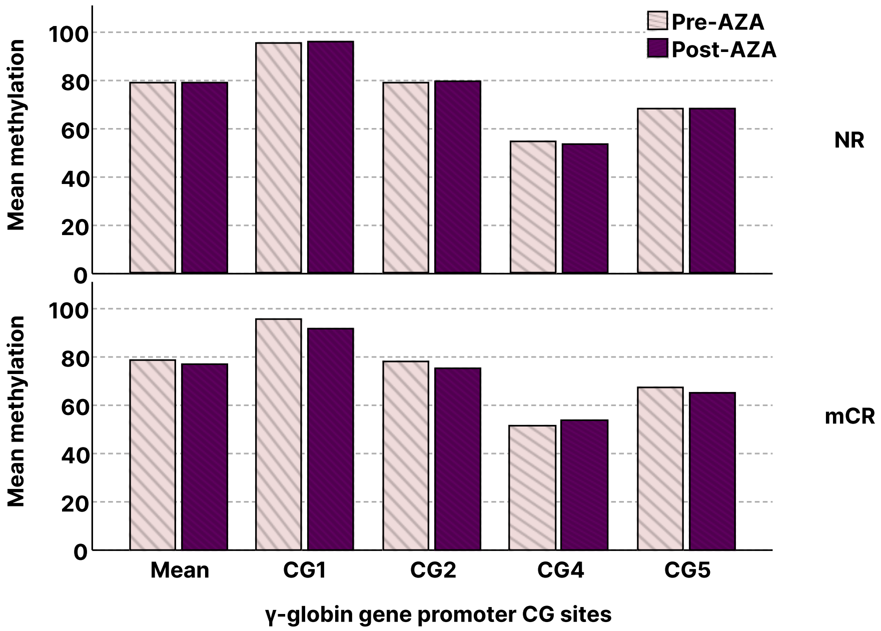

Figure S2. Methylation levels (%) of the γ-globin gene *(HBG2)* promoter at CG sites tested pre- and post-azacytidine (AZA) in mCR responders and NR patients.

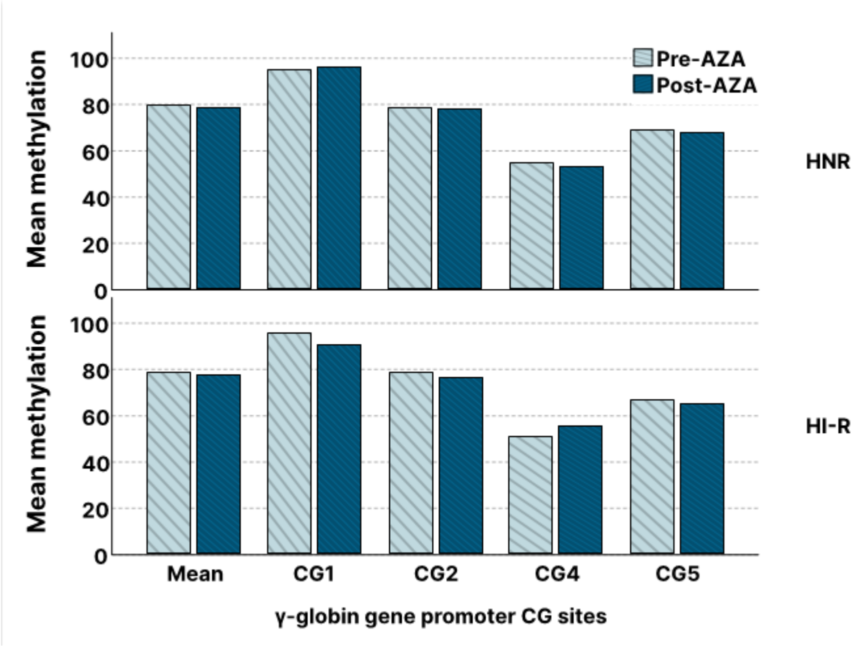

Figure S3.Methylation levels (%) of the γ-globin gene *(HBG2)* promoter at CG sites tested pre- and post-azacytidine (AZA) in HI-R responders and HNR patients.

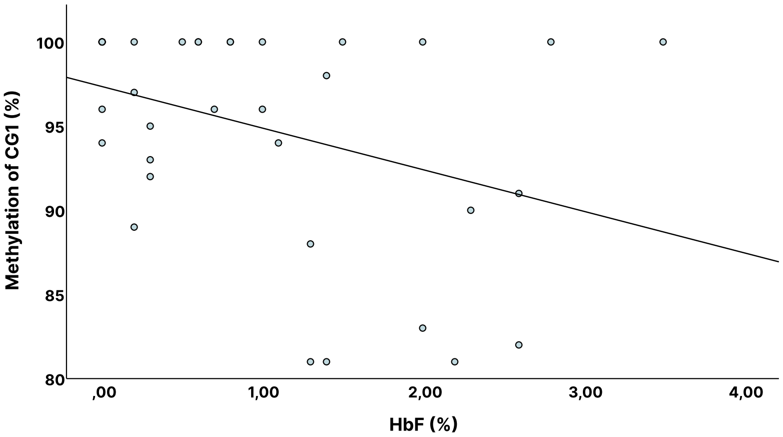

Figure S4. Methylation levels of CG1 of *HBG2* are related to HbF levels (all simultaneous methylation and HbF measurements included, both pre- and post-AZA, p_1=0.042, r_S=-0.341).

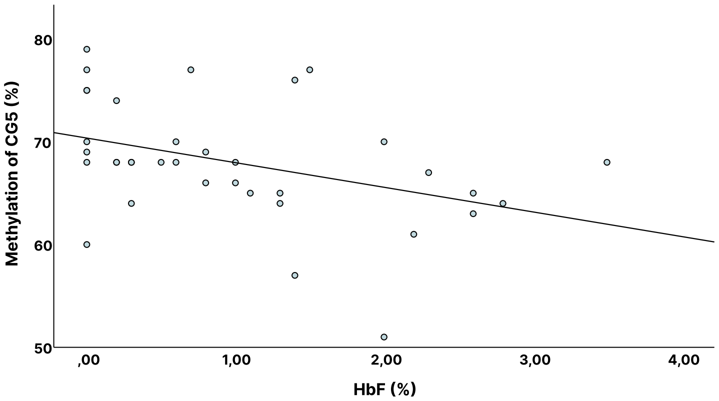

Figure S5. Methylation levels of CG5 of *HBG2* are related to HbF levels (all simultaneous methylation and HbF measurements included, both pre- and post-AZA, p_5=0.005, r_S=-0.461).

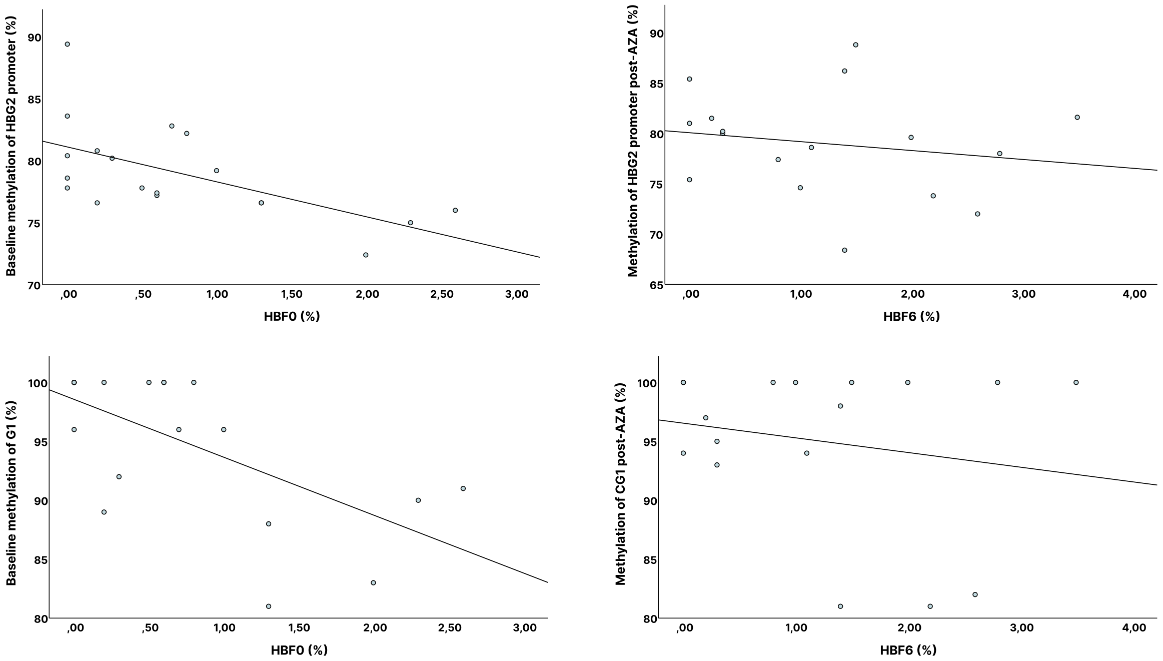

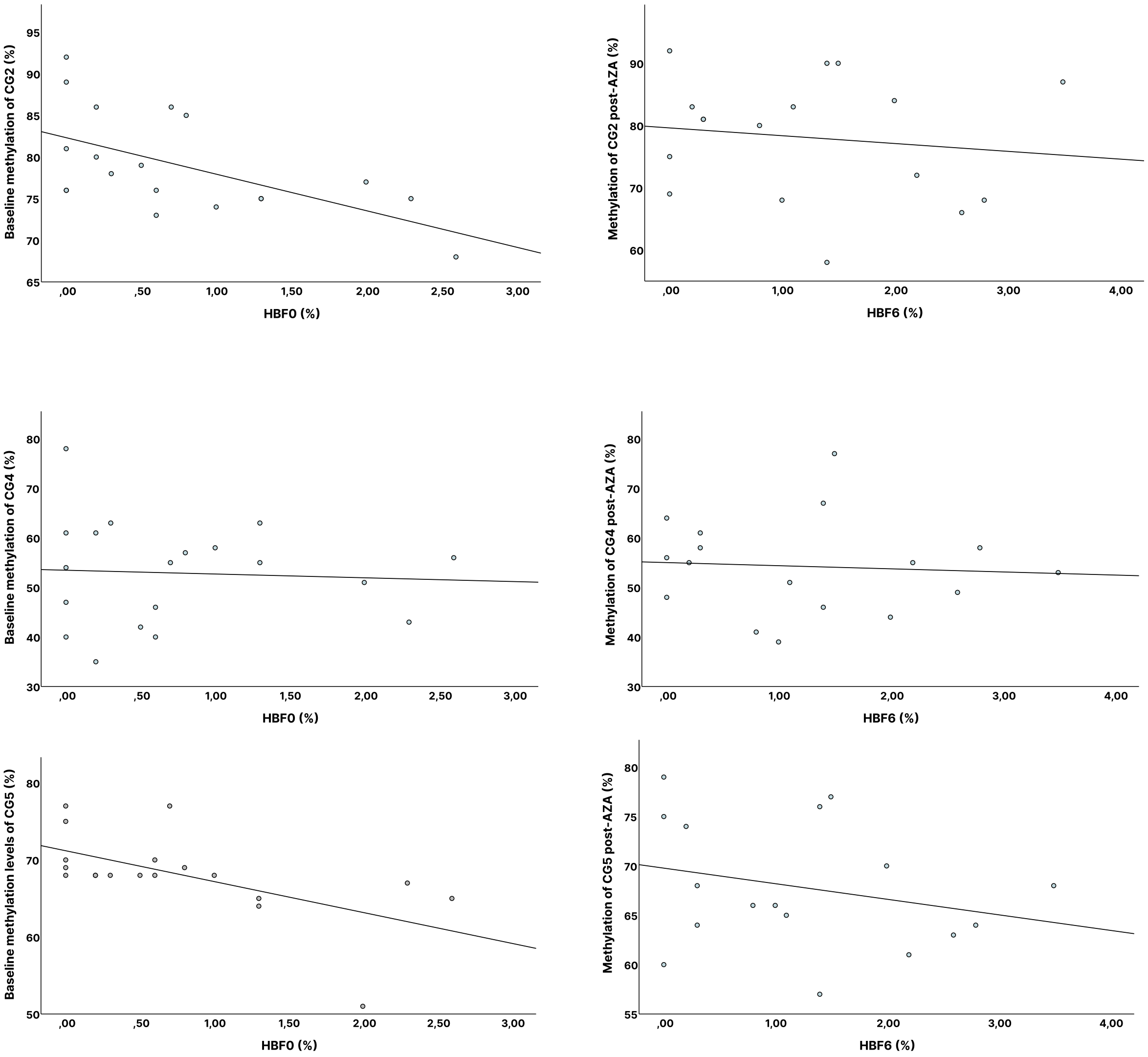

Figure S6. Scatter plots of CG sites of *HBG2* showing the relation between methylation and HbF levels pre- (baseline) and post-AZA (HbF0 and HbF6, respectively).

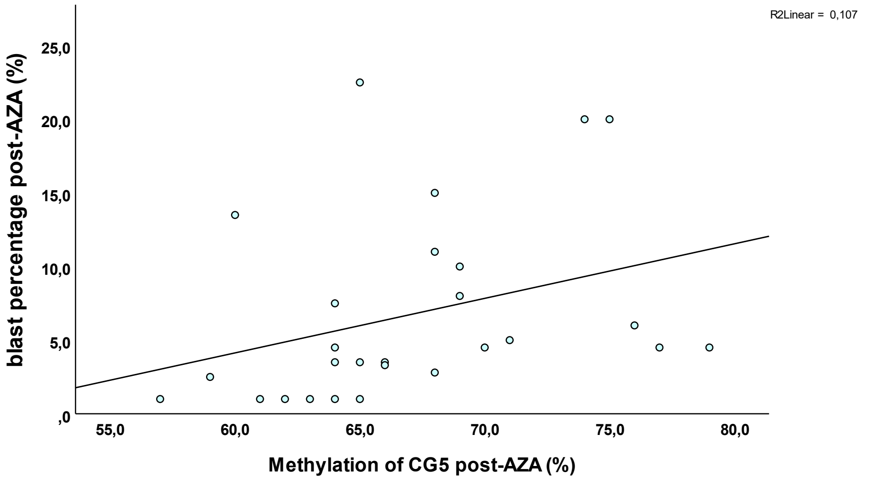

Figure S7. Lower methylation of CG5 of *HBG2* promoter post-AZA (after 5-7 cycles) is related to lower blast percentage post-treatment.
