# Supplementary material for: Fetal hemoglobin induction in azacytidine responders enlightens methylation patterns related to blast clearance in higher-risk MDS and CMML
Source: Clin Epigenetics. 2024 Jun 15;16:79. doi: 10.1186/s13148-024-01687-x (PMC11180405; doi:10.1186/s13148-024-01687-x)
Supplement: Supplementary file 4 — Supplementary tables and figures_2. [file 13148_2024_1687_MOESM4_ESM.pdf]

Fetal hemoglobin induction in azacytidine responders enlightens methylation patterns related to blast clearance in higher-risk MDS and CMML.

Theodora Chatzilygeroudi, Vasiliki Chondrou, Ruben Boers, Stavroula Siamoglou, Katerina Athanasopoulou, Evgenia Verigou, Joost Gribnau, Spyridon Alexis, Vassiliki Labropoulou, Alexandra Kourakli, George P. Patrinos, Argyro Sgourou, Argiris Symeonidis

Additional file 4: Supplementary tables and figures_2

Table S3. Genes with DMRs in patients treated with AZA and demonstration of response.

Patient ID	HMA given	Response	HbF regulating genes with changes in methylation patterns after AZA
A	AZA	CR	FOG-1, RCOR-1, ZBTB7A, MTA1, MTA2
B	AZA	mCR+HI	FOG-1, RCOR-1, ZBTB7A, BCL11A
C	AZA	mCR	FOG-1, ZBTB7A, MTA1, MTA2, MBD3, BCL11A
D	AZA	mCR+HI	No changes
E	AZA	NR	FOG-1, RCOR-1, ZBTB7A, MTA1, MDB3
F	AZA	NR	FOG-1, RCOR-1, ZBTB7A, MTA1, MTA2, MBD3
G	AZA	NR	No changes

NR: no response, mCR: marrow complete response, HI: hematological improvement, PR: partial response

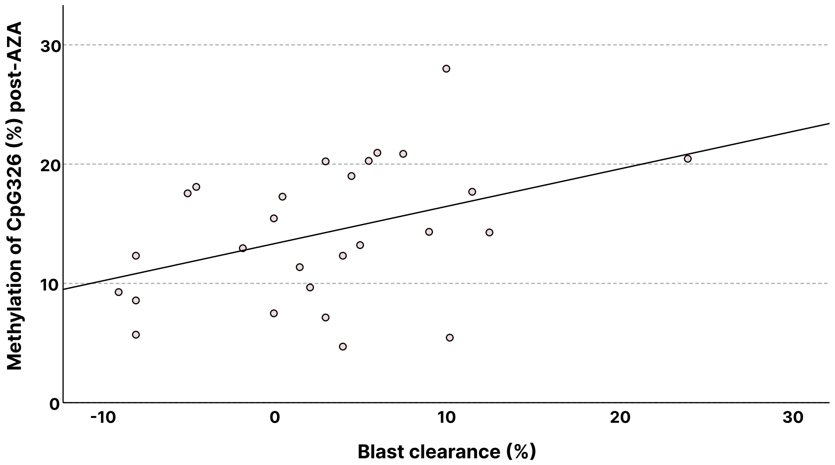

Figure S8. Methylation status of CpG326 island of *ZBTB7A* post-AZA treatment is related to blast cell clearance ($p=0.026$, $r_s=0.427$).

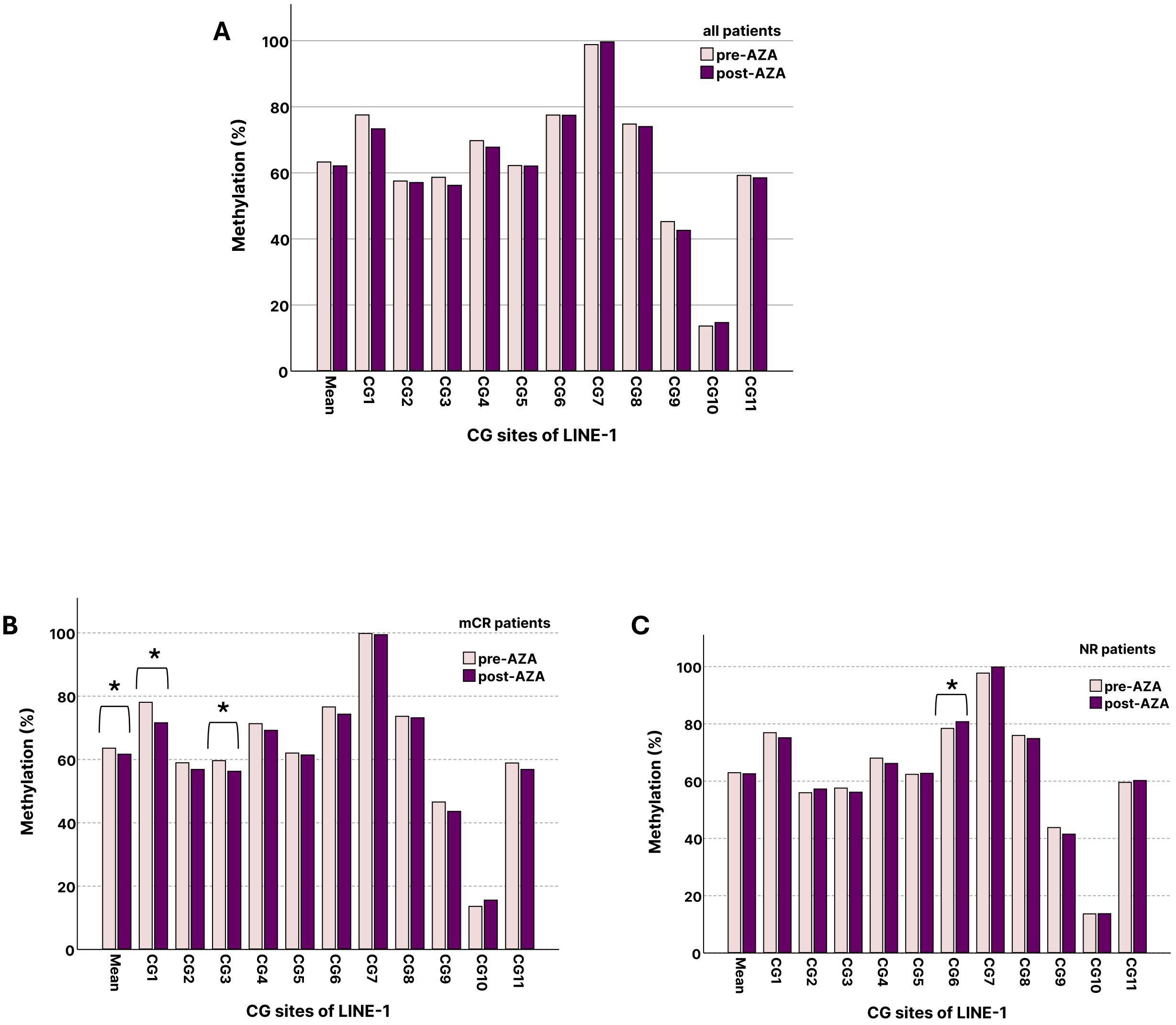

Figure S9. Methylation levels of LINE-1 CG sites tested pre- and post-AZA for A) all patients, B) marrow responders (mCR) and C) non-responding (NR) patients. Hypomethylation was observed only among responders (p_{mCR}=0.012) (B) and specifically at CG1 and CG3 sites (p_1=0.001, p_3=0.028). On the contrary, NR patients demonstrated hypermethylation of CG6 (p=0.044).

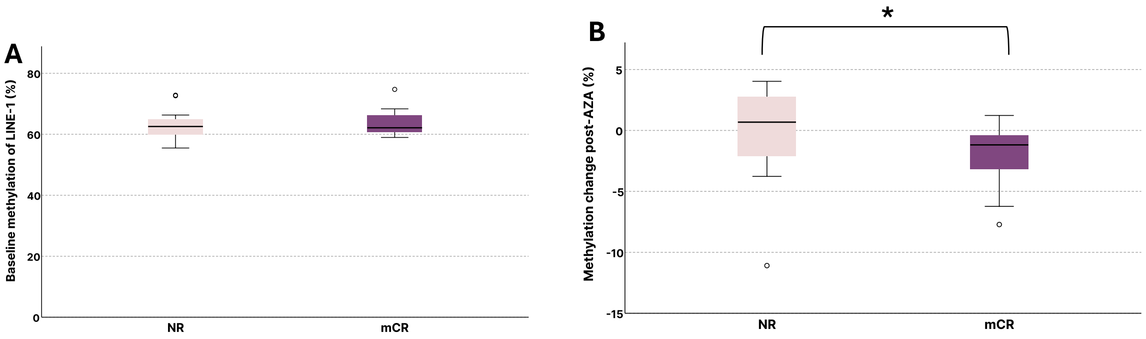

Figure S10. Methylation patterns of LINE-1 in AZA treated patients. A. No difference in pre-treatment LINE-1 methylation levels between mCR and NR patients (p=0.348). B. Methylation reduction post-AZA was greater in mCR responders (p=0.047).

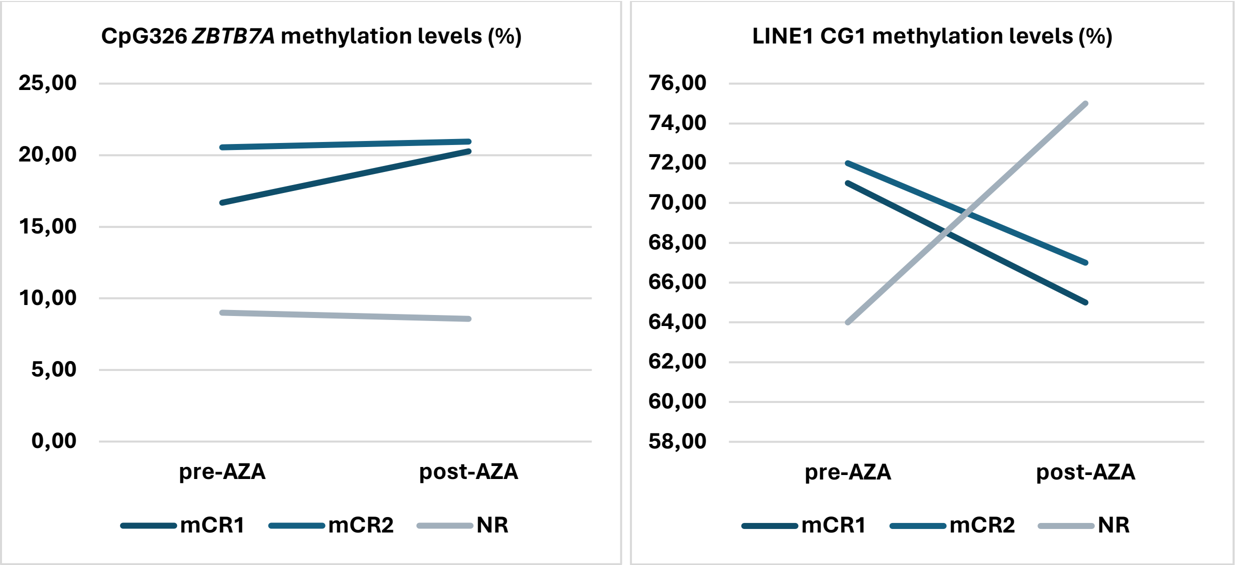

Figure S11. Methylation patterns of CpG326 of *ZBTB7A* and CG1 of LINE-1 in mCR and NR patients with stable cytogenetic/molecular status pre- and post-AZA.
