# Supplementary material for: Fetal hemoglobin induction in azacytidine responders enlightens methylation patterns related to blast clearance in higher-risk MDS and CMML
Source: Clin Epigenetics. 2024 Jun 15;16:79. doi: 10.1186/s13148-024-01687-x (PMC11180405; doi:10.1186/s13148-024-01687-x)
Supplement: Supplementary file 5 — Graphical presentation of significant differentially methylated regions (DMRs) of HbF regulating genes in high-risk MDS patients after treated with azacytidine (UCSC genome browser was used). [file 13148_2024_1687_MOESM5_ESM.pdf]

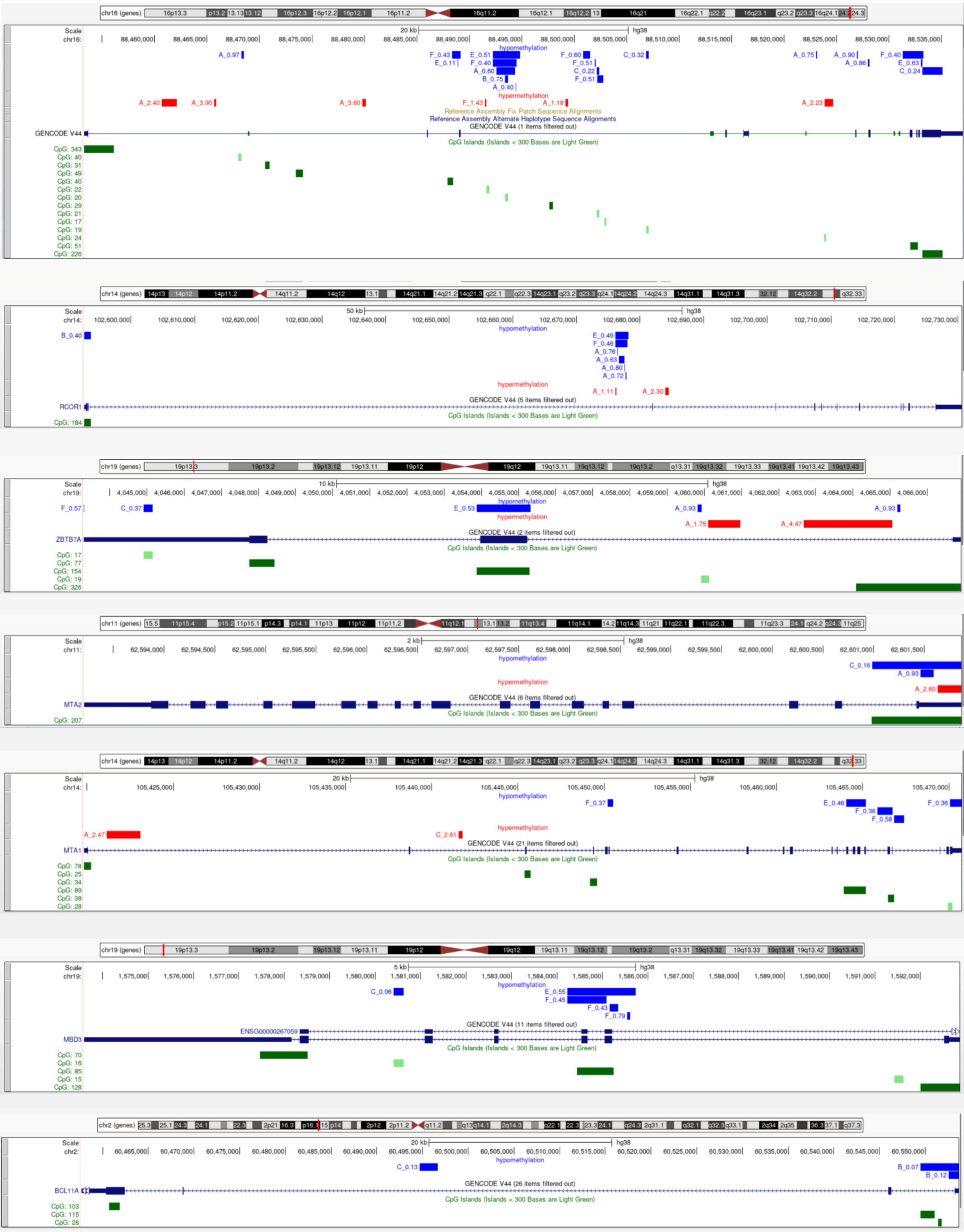
chr16 (genes)
Scale
chr16:
20 kb
hg38
hypomethylation
hypermethylation
Reference Assembly Fix Patch Sequence Alignments
Reference Assembly Alternate Haplotype Sequence Alignments
GENCODE V44 (1 items filtered out)
GENCODE V44
CpG Islands (Islands < 300 Bases are Light Green)
chr14 (genes)
chr14:
50 kb
GENCODE V44 (5 items filtered out)
RCOR1
chr19 (genes)
chr19:
10 kb
GENCODE V44 (2 items filtered out)
ZBTB7A
chr11 (genes)
chr11:
2 kb
GENCODE V44 (6 items filtered out)
MTA2
chr14 (genes)
chr14:
20 kb
GENCODE V44 (21 items filtered out)
MTA1
chr19 (genes)
chr19:
5 kb
GENCODE V44 (11 items filtered out)
ENSG00000267059
MBD3
chr2 (genes)
chr2:
20 kb
GENCODE V44 (26 items filtered out)
BCL11A
